# Tetrameric INTS6-SOSS1 complex facilitates DNA:RNA hybrid autoregulation at double-strand breaks

**DOI:** 10.1093/nar/gkae937

**Published:** 2024-10-24

**Authors:** Qilin Long, Kamal Ajit, Katerina Sedova, Vojtech Haluza, Richard Stefl, Sadat Dokaneheifard, Felipe Beckedorff, Monica G Valencia, Marek Sebesta, Ramin Shiekhattar, Monika Gullerova

**Affiliations:** Sir William Dunn School of Pathology, University of Oxford, South Parks Road, Oxford OX1 3RE, UK; Sir William Dunn School of Pathology, University of Oxford, South Parks Road, Oxford OX1 3RE, UK; CEITEC-Central European Institute of Technology, Masaryk University, Brno CZ-62500, Czech Republic; CEITEC-Central European Institute of Technology, Masaryk University, Brno CZ-62500, Czech Republic; CEITEC-Central European Institute of Technology, Masaryk University, Brno CZ-62500, Czech Republic; National Centre for Biomolecular Research, Faculty of Science, Masaryk University, Brno CZ-62500, Czech Republic; Department of Human Genetics, University of Miami, Miller School of Medicine, Sylvester Comprehensive Cancer Center, 1501 NW 10th Avenue, Miami, FL 33136, USA; Department of Human Genetics, University of Miami, Miller School of Medicine, Sylvester Comprehensive Cancer Center, 1501 NW 10th Avenue, Miami, FL 33136, USA; Department of Human Genetics, University of Miami, Miller School of Medicine, Sylvester Comprehensive Cancer Center, 1501 NW 10th Avenue, Miami, FL 33136, USA; CEITEC-Central European Institute of Technology, Masaryk University, Brno CZ-62500, Czech Republic; Department of Human Genetics, University of Miami, Miller School of Medicine, Sylvester Comprehensive Cancer Center, 1501 NW 10th Avenue, Miami, FL 33136, USA; Sir William Dunn School of Pathology, University of Oxford, South Parks Road, Oxford OX1 3RE, UK

## Abstract

DNA double-strand breaks (DSBs) represent a lethal form of DNA damage that can trigger cell death or initiate oncogenesis. The activity of RNA polymerase II (RNAPII) at the break site is required for efficient DSB repair. However, the regulatory mechanisms governing the transcription cycle at DSBs are not well understood. Here, we show that Integrator complex subunit 6 (INTS6) associates with the heterotrimeric sensor of ssDNA (SOSS1) complex (comprising INTS3, INIP and hSSB1) to form the tetrameric SOSS1 complex. INTS6 binds to DNA:RNA hybrids and promotes Protein Phosphatase 2A (PP2A) recruitment to DSBs, facilitating the dephosphorylation of RNAPII. Furthermore, INTS6 prevents the accumulation of damage-associated RNA transcripts (DARTs) and the stabilization of DNA:RNA hybrids at DSB sites. INTS6 interacts with and promotes the recruitment of senataxin (SETX) to DSBs, facilitating the resolution of DNA:RNA hybrids/R-loops. Our results underscore the significance of the tetrameric SOSS1 complex in the autoregulation of DNA:RNA hybrids and efficient DNA repair.

## Graphical Abstract

**Graphical Abstract:**
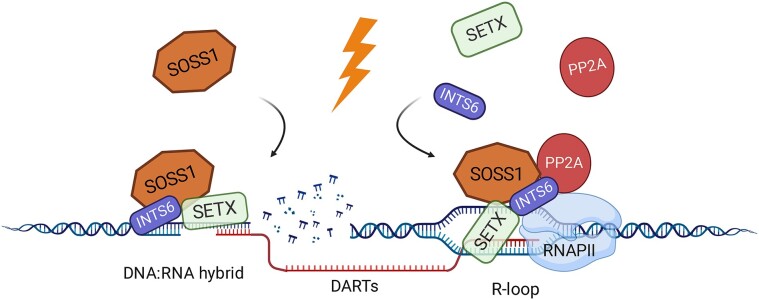

## Introduction

The stability of the human genome is challenged by thousands of DNA lesions, originating from both endogenous and exogenous sources (1). Double-strand breaks (DSBs) represent the most lethal form of DNA damage, disrupting the DNA double helix (2). In eukaryotes, there are two major repair pathways, homologous recombination (HR) and non-homologous end joining (NHEJ), to repair DSBs (1,3,4).

RNA polymerase II (RNAPII) is the enzyme that transcribes protein-coding genes and long non-coding RNAs (lncRNA). Unscheduled pausing of RNAPII may lead to the formation of R-loop structures, which consist of a DNA:RNA hybrid and a single stranded DNA (ssDNA) (5). R-loops, found behind paused RNAPII, are generally considered to be a by-product of transcription and a potential threat to genome stability due to the exposed ssDNA (6,7). Upon DNA damage, cells undergo transient global transcriptional repression, caused by physical blockage and/or degradation of RNAPII (8–11). Intriguingly, accumulating evidence suggests that temporary, damage-induced transcription at DSBs is required for efficient DNA repair (2,12–16). The *de novo* transcription at the DSBs leads to the production of nascent transcripts named damage-associated RNA transcripts (DARTs) or damage-induced long non-coding RNAs (dilncRNAs) (12,17,18). Both DARTs and dilncRNAs are derived from DSBs. DARTs are strand specific transcripts, generated at DSBs by RNAPII phosphorylated on its Y1 position (Y1P), which can be further distinguished as primary DARTs (pri-DARTs), initiating at the broken ends. These primary DARTs can hybridize to overhang ssDNA after resection, forming DNA:RNA hybrids, similarly to dilncRNA. Primary DARTs can also form R-loops behind paused RNAPII, which can in turn act as promoters of the secondary DARTs, which are transcribed towards DSBs (12). Both DARTs and dilncRNAs facilitate efficient DNA repair through the recruitment of other DNA damage response (DDR) factors (12,17–20). DNA:RNA hybrids and R-loops are predominantly formed at DSBs in transcriptionally active loci (21–24) and can facilitate the regulation of repair pathway choice (12,24–28). However, the prolonged existence of R-loops near DSBs can lead to genome instability (22,29,30).

The Integrator is a multi-protein complex (>1.5 MD), which consists of 16 subunits (INTS1-15 and DSS1/SEM1) (31), and regulates RNAPII activity (31–34). However, the understanding of the functional roles of Integrator’s individual subunits or sub-complexes is very limited. Integrator subunits INTS9 and INTS11 resemble CPSF-100 and CPSF-73, respectively, and exhibit similar functions in the cleavage of pre-mRNAs (31,34). Recently, INTS6, together with a non-canonical form of Protein Phosphatase 2A (PP2A), has been shown to form an Integrator–PP2A complex, which is recruited to actively transcribing genes to oppose CDK9 kinase activity and to dephosphorylate RNAPII (35–37). PP2A is a dominant serine-threonine phosphatase, which participates in numerous cellular processes in various tissues. Nevertheless, its function in regulating damage-induced transcription at DSBs remains elusive.

Notably, INTS3 and INTS6 play a role in DNA repair. INTS3, identified as a part of the heterotrimeric sensor of ssDNA (SOSS1) complex, along with hSSB1 (NABP2) and C9orf80 (INIP), contributes to efficient DNA repair (38). A similar complex consisting of INTS3 with INIP and hSSB2 (NABP1), named SOSS2, has also been implicated in DDR (39). INTS6 binds to the C-terminus of INTS3 *in vitro* (40), providing a scaffold for hSSB1/2 and INIP to form a tetrameric complex (41,42). However, the signals for the trimeric and tetrameric SOSS1/2 complex assembly, coordination between subunits, and their role in modulating transcription at DSBs remain unknown. We have recently showed that Abelson tyrosine kinase (c-Abl) phosphorylates hSSB1 as part of the trimeric SOSS1 complex which, together with RNAPII, promotes liquid–liquid phase separation at DSBs, enabling the formation of dynamic, transient compartments for DNA repair (38). Another study highlighted a stable association between the SOSS1 complex and the Integrator–PP2A complex to facilitate promoter-proximal termination of RNAPII transcription. The lack of SOSS1-Integrator–PP2A leads to increased RNAPII pausing and pervasive accumulation of R-loops, resulting in genome instability (43).

Senataxin (SETX) is a DNA:RNA helicase that resolves DNA:RNA hybrids in both damage and non-damage conditions (22,44,45). Mutations in SETX have been associated with multiple diseases, such as ataxia with oculomotor apraxia 2 or amyotrophic lateral sclerosis (46,47). Besides its roles in RNA processing, a recent study has revealed that SETX directly acts as a *bona-fide* RNAPII transcription termination factor (45). However, the precise mechanism governing the activity of SETX in DDR remains unclear.

In this study, we demonstrate that DNA damage stimulates the association of INTS6 with the trimeric SOSS1 complex to form the tetrameric complex, which subsequently recruits PP2A to DSBs. INTS6 alone, or as a part of tetrameric complex, binds to DNA:RNA hybrids. Furthermore, INTS6 interacts with SETX and facilitates its localization to damaged sites. The depletion of INTS6 results in increased levels of nascent DARTs/dilncRNAs and the accumulation of DNA:RNA hybrids at DSBs. Our data suggest that the coordinated activity of the INTS6-SOSS1-PP2A complex and SETX drives the autoregulation of DNA:RNA hybrids, thus promoting efficient DNA repair.

## Materials and methods

### Plasmids

The Open Reading Frame (ORF) of INTS6 was cloned into plasmid 438C (pFastBac His6 MBP Asn10 TEV cloning vector with BioBrick Polypromoter LIC subcloning, Addgene plasmid #55220). Constructs 438B-INTS3, 438B-hSSB1, 438B-INIP and 438C-INTS6 were combined using BioBrick Polypromoter LIC subcloning into a single construct enabling co-expression of the four subunits of the tetrameric INTS6-SOSS1 complex from a single virus in insect cells. To generate plasmids enabling expression of the kinase module of TFIIH complex in insect cells, the ORFs for CDK7, MAT1 and CCNH were cloned into plasmid 438B and later combined into a single construct. Plasmid enabling expression of cABL^CAT^ (AA83–534), alongside PTP1b^1-238^, was generously provided by Gabriele Fendrich and Michael Becker at the Novartis Institutes for Biomedical Research, Basel. Plasmid pGEX4T1–(CTD)_26_–(His)_7_ (provided by Olga Jasnovidova) was used to express and purify GST–(CTD)_26_–(His)_7_.

### Insect cell work

To generate viruses enabling the production of proteins in insect cells, the coding sequences and the necessary regulatory sequences of the constructs were transposed into bacmid using *Escherichia coli* strain DH10bac. The viral particles were obtained by transfection of the bacmids into the *Sf*9 cells using FuGENE Transfection Reagent and further amplification. Proteins were expressed in 600 ml of Hi5 cells (infected at 1 × 10^6^ cells/ml) with the corresponding P1 virus at multiplicity of infection >1. The cells were harvested 48 h post-infection, washed with 1× phosphate-buffered saline (PBS) and stored at −80°C.

### Protein purification

#### Purification of MBP-INTS6 and MBP-INTS6-SOSS complex

Pellets of Hi5 insect cells were resuspended in ice-cold lysis buffer [50 mM Tris (pH = 8.0); 500 mM NaCl; 0.4% Triton X-100; 10% (v/v) glycerol; 10 mM imidazole; 1 mM Dithiothreitol (DTT); protease inhibitors (0.66 μg/ml pepstatin, 5 μg/ml benzamidine, 4.75 μg/ml leupeptin, 2 μg/ml aprotinin); and 25 U benzonase per ml of lysate]. The resuspended cells were gently shaken for 10 min at 4°C. To aid the lysis, cells were briefly sonicated. The cleared lysate was passed through 2 ml of Ni-NTA beads (QIAGEN), equilibrated with buffer [50 mM Tris-HCl (pH = 8); 500 mM NaCl; 10 mM imidazole; and 1 mM DTT]. Proteins were eluted with an elution buffer [50 mM Tris-HCl (pH = 8); 500 mM NaCl; 1 mM DTT; and 400 mM imidazole]. The elution fractions containing proteins were pooled, concentrated and further fractioned on Superdex S-200 column (for MBP-INTS6 purification) or Superose 6 column (for INTS6-tetrameric complex purification) with SEC buffer [25 mM Tris-Cl (pH = 7.5); 200 mM NaCl, 1 mM DTT]. Fractions were then concentrated, and glycerol was added to a final concentration of 10% before they were snap-frozen in liquid nitrogen, and stored at −80°C.

#### Purification of GST-CTD and mGFP-CTD

Five grams of *E. coli* BL21 RIPL cells expressing GST–(CTD)_26_–(His)_7_ and mGFP–hCTD were resuspended in ice-cold lysis buffer [50 mM Tris-HCl, (pH = 8); 0.5 M NaCl; 10 mM imidazole; 1 mM DTT], containing protease inhibitors (0.66 μg/ml pepstatin, 5 μg/ml benzamidine, 4.75 μg/ml leupeptin, 2 μg/ml aprotinin) at +4°C. Cells were opened up by sonication. The cleared lysate was passed through 2 ml of Ni-NTA beads (QIAGEN) and equilibrated with buffer [50 mM Tris-HCl (pH = 8); 500 mM NaCl; 10 mM imidazole; and 1 mM DTT]. The proteins were eluted with an elution buffer [50 mM Tris-HCl (pH = 8); 500 mM NaCl; 1 mM DTT; and 400 mM imidazole]. The elution fractions containing CTD peptides were pooled, concentrated and further fractioned on Superdex S-200 column with SEC buffer [25 mM Tris-Cl (pH = 7.5); 200 mM NaCl; 1 mM DTT]. Fractions containing pure CTD polypeptides were concentrated, glycerol was added to a final concentration of 10% before they were snap-frozen in liquid nitrogen and stored at −80°C.

#### Preparative phosphorylation and purification of CTD polypeptides

For preparative purposes, 2.5 mg of GST-(CTD)_26_–(His)_7_ and mGFP–hCTD, respectively, were phosphorylated by 250 μg of the CDK7 complex (to phosphorylate serine 5 and serine 7 on the CTD) and by 250 μg of c-Abl (to phosphorylate tyrosine 1 on the CTD), respectively, in the presence of 2 mM ATP and 3.5 mM MgCl_2_ for 60 min at 30°C. Reactions were stopped by placing the mixtures at +4°C. The CTD peptides were purified from the kinases and ATP by size-exclusion chromatography on Superdex S-200, equilibrated with 25 mM Tris-Cl (pH = 7.5), 220 mM NaCl and 1 mM DTT. Fractions containing phosphorylated CTD polypeptides were pooled, concentrated, snap-frozen in liquid nitrogen and stored at −80°C.

### Electrophoretic mobility shift assay

Increasing concentrations of the tested proteins (22, 44, 88, 167 nM) were incubated with fluorescently labeled nucleic acid substrates (final concentration 10 nM) in buffer D [25 mM Tris-Cl (pH = 7.5); 1 mM DTT; 5 mM MgCl_2_; and 100 mM NaCl] for 20 min at 37°C. Loading buffer [60% glycerol in 0.001% Orange-G] was added to the reaction mixtures and the samples were loaded onto a 7.5% (w/v) polyacrylamide native gel in 0.5× TBE buffer and run at 75 V for 1 h at +4°C. The different nucleic acid species were visualized using an FLA-9000 Starion scanner and quantified in the MultiGauge software (Fujifilm). To calculate the relative amount of bound nucleic acid substrate, the background signal from the control sample (without protein) was subtracted using the *band intensity – background* option. Nucleic acid-binding affinity graphs were generated with Prism-GraphPad 7.

### *In vitro* pull-down experiments

Purified GST, GST-CTD, GST-Y1P-CTD and GST-S5,7P-CTD (5 μg each), respectively, were incubated with the tetrameric SOSS1 complex and MBP-INTS6, respectively (5 μg each) in 30 μl of buffer T [20 mM Tris-Cl, 200 mM NaCl, 10% glycerol, 1 mM DTT, 0.5 mM ethylenediaminetetraacetic acid (EDTA) and 0.01% Nonidet *P*-40; pH = 7.5] for 30 min at 4°C in the presence of GSH-beads. After washing the beads twice with 100 μl of buffer T, the bound proteins were eluted with 30 μl of 4× SDS loading dye. The input, supernatant and eluate, 7 μl each, were analyzed on SDS-polyacrylamide gel electrophoresis gel.

### Micro-scale thermophoresis

Binding affinity comparisons via microscale thermophoresis were performed using the Monolith NT.115 instrument (NanoTemper Technologies). The CTD polypeptides (CTD, Y1P-CTD and S5,7P CTD, respectively) were fused with msfGFP and served as ligands in the assays. Affinity measurements were performed in the micro-scale thermophoresis (MST) buffer [25 mM Tris-Cl buffer (pH = 7.5); 150 mM NaCl; 1 mM DTT; 5% glycerol; and 0.01% Tween-20]. Samples were soaked into standard capillaries (NanoTemper Technologies). Measurements were performed at 25°C, 50% LED, medium IR-laser power [laser on times were set at 3 s before MST (20 s) and 1 s after], constant concentration of the labeled ligand (20 nM), and increasing concentration of the trimeric SOSS complex (4.8–1200 nM, CTD-GFP and Y1P-CTD-GFP; 28.7–7250 nM, S5,7P CTD) or the tetrameric SOSS complex (3.4–846 nM, CTD-GFP and Y1P-CTD-GFP; 4.3–607.5 nM, S5,7P CTD), respectively. The data were fitted with Specific binding Hill Slope in GraphPad Prism software.

### Cell lines and cell culture

Cells were maintained in high-glucose Dulbecco's modified Eagle’s medium (DMEM) medium (Life Technologies, 31966047) with 10% (v/v) fetal bovine serum (Merck, F9665-500ml), 2 mM L-glutamine (Life Technologies, 25030024) and 100 units/ml penicillin–streptomycin solution (Life Technologies, 15140122) at 37°C with 5% CO_2_ supplement. The frequent mycoplasma test was conducted, and regular cell morphology authorization was performed with microscope. HeLa wild-type (WT) cells were obtained from ATCC. WT U2OS or *Asi*SI-ER U2OS cells are gifts from the Legube Laboratory (CNRS – University of Toulouse, France).

### Chemicals, antibodies and damage induction

Ionizing radiation (IR)-induced DNA damage was performed by using the CS-137 source (Gravatom, GRAVITRON RX30/55). Generally, IR = 10 Gy, and samples were harvested 10 min post-IR unless stated differently. Cells were incubated with 20 μM triptolide (TPL) (Enzo Life Sciences, BV-1761–1) for 1 h or 100 μM 5,6-dichloro-1-beta-D-ribofuranosylbenzimidazole (DRB) (Cayman Chemical, 10010302) for 2 h or 1 μM THZ1 (Stratech Scientific, A8882-APE-10mM) for 2 h prior to the induction of DNA damage; 2.5 μM LB-100 (Stratech Scientific, B4846-APE-5mg) was employed for 2 h to inhibit PP2A. The break induction in *AsiSI*-ER U2OS cells was achieved by using 400 nM (Z)-4-hydroxytamoxifen (4OHT) (Cayman Chemical, 14854–1mg-CAY) for 4 h. For ChIP experiment, 2.5 μM LB-100 and 400 nM 4OHT were added to cells together and cells were incubated for 4 h. For HeLa NHEJ reporter cells, the selective DNA-PK inhibitor BAY-8400 (Cambridge Bioscience, HY-132293–1mg, 2 μM) was used as a control. The used antibodies were listed in Supplementary Table S5.

### RNA interference and plasmid transfection

RNA interference (25 nM for siRAD51, 60 nM for the rest of small interfering RNA (siRNAs) used in this study) was used with Lipofectamine RNAiMAX (Life Technologies, 13778075) in OPTI-MEM (Gibco, 11058021) by reverse transfection method for 48 h. The siRNAs used in this study are listed in the Supplementary Table S5. The forward transfection method was used to deliver plasmids with Lipofectamine 3000 (Invitrogen, L3000001) or Lipofectamine LTX (Invitrogen, 15338100). Gibson cloning was used to generate INTS6-GFP plasmid. DNA fragment (CDS) of INTS6 from IDT was inserted into pCMV3-C-GFPSpark^®^ backbone (Sino Biological, HG22790-ACG) [polymerase chain reaction (PCR) amplification with primers listed in Supplementary Table S3] with NEBuilder HiFi DNA Assembly Cloning Kit (NEB, E5520S). Plasmid sequence was confirmed by Sanger sequencing.

### Western blot

Cells were lifted by trypsin (Life Technologies, 12604013) and resuspended with 1× Laemmli buffer [62.5 mM Tris (pH = 6.8), 2% sodium dodecyl sulphate (SDS), 2% β-mercaptoethanol, 10% glycerol, 0.005% bromophenol blue] (Alfa Aesar, J61337AD) and denatured for 10 min at 95°C. Sonication step (high power, 10 s with probe sonicator) was included to reach a complete cell lysis. Each sample was centrifuged at 15 000 g for 15 min to pellet cell debris before loading samples onto a gel. NuPAGE™ 4–12%, Bis-Tris, 1.0 mm, Midi Protein Gels (Invitrogen™, WG1402BOX) (for SETX detection) and 4–15% Mini-PROTEAN^®^ TGX™ precast protein gels (BioRad, 4561083/4561086) (for the rest proteins) were used with standard western blot protocol. Briefly, gel was electrophoresed in MOPS running buffer (for NuPAGE Bis-Tris gel) or Tris-Glycine running buffer (for PROTEAN^®^ TGX™ gel) at 120 V until proteins were separated. Subsequently, proteins on PROTEAN^®^ TGX™ gel were transferred onto nitrocellulose membranes (Perkinelmer, NBA085A001EA) with Trans-Blot^®^ Turbo™ Transfer System (Bio-Rad, 1704150) at 25 V, constant 1.8 A for 10 min. For proteins on NuPAGE™ 4–12% Bis-Tris gel, wet transfer at 280 mA for 2.5 h was employed. Membranes were then blocked with 5% milk (Sigma, 70166–500G) in PBS with 1% TWEEN-20 (Fisher Scientific, BP337-100) (PBST) at room temperature for 1 h. Primary antibodies listed in the Supplementary Table S5 were incubated overnight at 4°C. Proper secondary antibodies were used for 1 h at room temperature before visualized on the membranes with Pierce™ ECL Western Blotting Substrate (Thermo Scientific, 10005943) or SuperSignal™ West Pico PLUS Chemiluminescent Substrate (Thermo Scientific, 34580) and Amersham™ Hyperfilm™ ECL™ (VWR, 28–9068-35) film.

### Proximity ligation assay

Proximity ligation assay (PLA) was performed by using Duolink™ In Situ Red Starter Kit Mouse/Rabbit (Merck, DUO92101-1KT) according to the manufacturer’s instructions; 2 × 10^5^ cells were seeded onto glass coverslip (SLS, MIC3300) overnight before fixed with 4% paraformaldehyde (PFA) in PBS (Alfa Aesar, J61899) for 10 min. After washing away 4% PFA with ice-cold PBS (five times), permeabilization step was performed with 0.1% Triton X-100 (Merck, X100-100ML) in PBS for 10 min before blocking (inside a humanity chamber) with 100 μl blocking buffer from the kit for 1 h at 37°C. The primary antibody was diluted with Duolink™ dilution buffer and incubated on coverslips overnight at 4°C. Following primary antibody incubation, PLA probe incubation, ligation and amplification processes were followed by the manufacturer's instructions. Coverslips were mounted with DAPI solution and sealed onto clear slides and air-dry in dark before imaging with Olympus FluoView Spectral FV1200 confocal microscope with 60× oil immersion objective. Images were processed in FIJI software (48) quantified by using CellProfiler (49) 4.2.1 with sparkle counter pipeline.

### Laser microirradiation

A total of 1 × 10^6^ U2OS cells were reverse transfected with INTS6-GFP alone or together with pFRT-TODestRFP RNAseH1 (Addgene, 65785) for 24 h and re-seeded (2 × 10^5^ cells) onto CELLview Culture dish (35 mm) (Greiner, 627860). After 24 h, 10 μM Hoescht 33342 (Thermo Scientific, H3570) was used to sensitize cells for 30 min. The laser microirradiation was performed with Nikon SoRa microscope, whilst cells were maintained at 37°C and 5% CO_2_ during the experimental procedures. Laser stripes were made by a 405 pulsed laser with laser power set to 10% at 20 repetitions. The frames using 488 and 555 nm channels were acquired every 4 s. The images were processed using FIJI software (48). The relative green fluorescent protein (GFP) or red fluorescent protein (RFP) intensity was quantified, as measured at the laser stripe with background bleaching being subtracted.

### Cell lysis and co-immunoprecipitation

Approximately 1 × 10^7^ cells in a 15 cm dish (at 50–70% confluency) were washed twice with PBS before being collected by spinning (500 g, 4°C, 5 min) in a 1.5 ml tube. The cell pellet was then lysed in 5× volumes of Lysis buffer (300 μl) [50 mM Tris (pH = 8) (Merck, T6066); 150 mM NaCl (Merck, S3014); 2.5 mM MgCl_2_ (Merck, PHR2486); 1% NP40 (Merck, I8896-100ML); 10% glycerol (Thermo, 032450.M1); 1× protease inhibitors (Merck, 11873580001)/1× phosphatase inhibitors (PPI; Thermo Fisher, A32961)] and 1 μl/sample of Benzonase^®^ Nuclease (Merck, E1014-25KU) was incubated on a wheel for 1 h at 4°C with vigorous mixing every 15 min. The cell lysate was subsequently collected by spin at 13 000 revolutions per minute (rpm) at 4°C for 10 min before being diluted with 1.5× cell volumes (450 μl) of dilution buffer (150 mM NaCl, 2.5 mM MgCl_2_, 10% glycerol, 1× PPI) inside a fresh 2 ml tube; 0.05–0.1× volume of diluted cell lysate was taken as input. The Dynabeads Protein A (Life technologies, 10002D) or Dynabeads Protein G (Life technologies, 10004D) were blocked with 3% bovine serum albumin (BSA) in dilution buffer before being added to cell lysate for 1 h at 4°C with rotation (25 μl/sample beads resuspended in 25 μl/sample dilution buffer). The pre-cleared cell lysate was incubated with antibody at 4°C overnight. The pulldown next day was performed with 25 μl/sample Protein A/G agarose beads for 1.5 h at 4°C. Then, the beads were washed with dilution buffer three times before being eluted with 2× Laemmli buffer and denatured for 10 min at 95°C. For protein fused to GFP, GFP-Trap magnetic agarose beads (Proteintech, gtma-20) were used for a pull down. Cell lysis was prepared as above and rotated at 4°C for 1 h before washing and elution steps were performed.

### Affinity purification of FLAG-tagged INTS6 or mock for mass spectroscopy

HEK293 cells overexpressing stable FLAG-tagged INTS6 (FLAG-INTS6), or mock were cultured in DMEM media (Gibco, #11965–084) supplemented with puromycin and 10% Fetal bovine serum (FBS) (Atlas Biologicals, #F-0500-D). The purification of nuclear FLAG-INTS6 or mock was performed as described in Kirstein *et al.* (50). Briefly, the removal of the cytoplasmic fraction and nuclear lysates were extracted using 10 ml of the buffer containing 0.42 M NaCl, 20 mM Tris-HCl (pH = 7.9), 1.5 mM MgCl2, 0.5 mM DTT, 25% glycerol, 0.2 mM EDTA and 0.2 mM phenylmethylsulfonyl fluoride (PMSF). The complex was purified using its incubation with 1 ml of anti-FLAG M2 affinity gel (Sigma) 6h at 4°C. Following spinning down the vial at 2000 g for 2 min, pellet was washed twice with 10 ml of buffer BC500 [20 mM Tris (pH = 7.6), 0.01% Triton X-100, 0.2 mM ETDA, 10 mM 2-mercaptoethanol, 10% glycerol, 0.2 mM PMSF and 0.5 M KCl], and four times with 10 ml of the buffer BC100 (0.01% Triton X-100, 20 mM Tris (pH = 7.6), 10% glycerol, 0.2 mM EDTA, 100 mM KCl, 10 mM 2-mercaptoethanol and 0.2 mM PMSF) and one final wash of BC100 without detergent. Following washing, affinity columns were eluted with 500 μl of FLAG peptide solution (0.5 μg/μl) resuspended in BC100 buffer.

The silver staining was performed as described in Kirstein *et al.* (50). Briefly, nuclear lysates of inputs and affinity-purified elution were loaded on a 4–20% Tris-glycine gel (Invitrogen, Cat# XP04205BOX), 10% acetic acid and 50% methanol was used to fix the gel in for 1 h at room temperature. To complete fixation, the gel was transferred to 10% methanol and 7% acetic acid for 1 h. For washing the gel, 10% glutaraldehyde was used for 15 min, followed by three times washing in MilliQ water for 15 min. Gel was stained for 15 min in 100 ml of staining solution [1 g AgNO_3_, 2.8 ml NH4OH, 185 μl NaOH (stock 10N in MilliQ water), brought it up to 100 ml with MilliQ water]. After washing three times for 2 min in MilliQ water, the gel was developed in 100 ml developing solution (0.5 ml 1% citric acid and 52 μl 37% formaldehyde in 100 ml MilliQ water). A solution containing 5% acetic acid and 50% methanol was used to stop the reaction.

Nuclear lysates of inputs and affinity-purified elution were loaded onto 4–15% Criterion TGX Stain-Free precast polyacrylamide gels (Bio-Rad, Cat# 5678085) and transferred to nitrocellulose membranes which were subsequently blocked by 5% BSA for 1 h at room temperature. Membranes were incubated with primary antibodies [including: anti-INTS6 (R90 N-terminal homemade antibody), anti-INTS11 (Sigma Prestige, #HPA029025), anti-Senataxin (Abcam, 300439), anti-PP2A-C (CST, #2259), anti-PP2A-A (CST, #2041) and anti-RPB1 NTD (CST, #14958) for overnight at 4°C]. Then following three times washing for 10 min, blots were incubated with horseradish peroxidase-conjugated secondary antibodies for 30 min at room temperature. Western blot results were visualized and quantitated by iBright 1500 Imaging system.

### Chromatin immunoprecipitation

Chromatin immunoprecipitation (ChIP) and quantitative polymerase chain reaction (qPCR) were performed by standard procedures as previously described (12). After the 4 h DSB induction by 4OHT, 1 × 10^7^ WT U2OS or *Asi*SI-ER U2OS cells were crosslinked with 1% formaldehyde (Merck, 252549–100ML) for 10 min at 37°C and inactivated by the addition of glycine (Merck, G7126) to a final concentration of 125 mM for 10 min (at 37°C). Cells were detached with a cell lifter (Fisher Scientific, 11577692) and washed with ice-cold PBS twice (by spin 5 min, 400 g). The cell pellet was lysed with 500 μl of cell lysis buffer [5 mM PIPES (VWR, 0169–100g), 85 mM KCl, 0.5% NP-40, 1× PPI] on ice for 10 min. The samples were then centrifuged at 800 g for 5 min to remove cytoplasm fractions (supernatant). Nuclei pellet was resuspended with 400 μl nuclear lysis buffer [50 mM Tris–HCl (pH = 8.0), 1% SDS (Merck,75746–1KG), 10 mM EDTA, 1× PPI] and incubated on ice for another 10 min. Following nuclei lysis, chromatins were sheared by Bioruptor^®^ Pico (Diagenode) for 10 min (high power, 30 s ON/OFF) to reach ∼500 bp length. The supernatants containing the sheared chromatin were collected by centrifugation (14 000 g, 4°C, 10 min) and diluted with 2.5× volumes (∼1 ml) dilution buffer [16.7 mM Tris-HCl (pH = 8.0), 0.01% SDS, 1.1% Triton X-100, 5 mM EDTA, 167 mM NaCl, 1× PPI] and precleared by 30 μl protein A/G agarose beads (Merck-Millipore, 16–157/16–201) for 1 h. A total of 5 μg of antibody was used to isolate protein–DNA complex overnight at 4°C (with rotation). The protein–DNA complex was pulled down by 40 μl protein A/G agarose beads for 1.5 h at 4°C and washed with buffer A [20 mM Tris-HCl (pH = 8.0); 2 mM EDTA; 1% SDS; 0.1% Triton X-100; and 150 mM NaCl] once, buffer B [20 mM Tris-HCl (pH = 8.0); 2 mM EDTA; 0.1% SDS; 1% Triton X-100; and 500 mM NaCl] once, buffer C [10 mM Tris-HCl (pH = 8.0); 1 mM EDTA; 1% NP-40; 1% sodium deoxycholate (DOC) (Merck, D6750-100G); and 250 mM LiCl (Merck, L4408-100g)] once and buffer D [10 mM Tris-HCl (pH = 8.0) and 1 mM EDTA] twice. Then, protein–DNA complex was eluted from beads with elution buffer [1% SDS and 100 mM NaHCO_3_] by rotating at room temperature for 30 min. To free DNA from protein–DNA complex, 55 μl digest buffer [400 mM Tris-HCl (pH = 6.5) and 100 mM EDTA], 30 μl 5 M NaCl (to reach 300 mM), 1 μl RNase A (10 μg/ml) (Thermo Scientific™, EN0531) and 2 μl Proteinase K (10 mg/ml) (Thermo Scientific™, EO0491) were added to sample tube and incubated at 65°C overnight. DNA was purified by phenol/chloroform (pH = 7.0) (Thermo Fisher, 10308293) and ethanol precipitation. qPCR was performed in triplicate using 1 ng isolated genomic DNA in a 25 μl reaction containing SensiMix™ SYBR^®^ (Scientific Laboratory Supplies, QT65005) and 10 μM each of forward and reverse primers (listed in Supplementary Table S2) on Rotor-Gene Q (QIAGEN) with PCR procedures under the following programme: 1 cycle at 95°C for 10 min; 45 cycles at 95°C, 15 s and 62°C for 15 s; 1 cycle at 72°C for 20 s. The 2^ΔΔCt^ method was applied for quantification. Data are represented as mean ± standard deviation (SD).

### DNA:RNA hybrid immunoprecipitation

To preserve the native DNA:RNA hybrid structures, all the procedures were performed in cold room. The non-crosslinked *Asi*SI cells (5 × 10^6^ −1 × 10^7^) were trypsinized and collected before incubated with 800 μl cell lysis buffer [85 mM KCl; 5 mM PIPES; and 0.5% NP-40] for 10 min. After cell lysis, samples were spined at 500 g, 4°C for 5 min to pellet nuclei fractions and remove the cytoplasmic supernatant. The nuclei pellet was lysed in 800 μl nuclei lysis buffer [50 mM Tris-HCl (pH = 8.0); 5 mM EDTA; and 1% SDS] for 10 min before being subjected to Proteinase K digestion (10 μl, 4 h). Initially, 5 μl Proteinase K (10 mg/ml) (Thermo Scientific™, EO0491) were used and incubated at 55°C for 1 h with vigorous pipetting every 15 min. After that, an additional 3 μl of Proteinase K was added to each tube for another 1 h at 55°C incubation. Another 2 μl of Proteinase K was added to each tube for additional 2 h incubation (55°C). Chromatin were subsequently precipitated by 5 M KAc and isopropanol, washed with 75% ethanol. After the air-dry of chromatin, 100 μl diethyl pyrocarbonate (DEPC) H_2_O was used to resuspend chromatin at room temperature for 3 min and diluted with 300 μl immunoprecipitation (IP) dilution buffer [16.7 mM Tris-HCl (pH = 8.0); 0.01% SDS; 1.1% Triton X-100; 1.2 mM EDTA; and 167 mM NaCl, 1× PPI] before being sonicated by Bioruptor (Diagenode, medium power, 30 s on, 30 s off for 10 min). Samples were precleared with 25 μl/sample Protein A Dynabeads (Life Technologies, 10002D) for 1 h before incubated with 2.5 μg S9.6 antibody (Sigma, MABE1095) for 10 h at 4 °C. The pull-down of S9.6-DNA:RNA hybrids was achieved by 25 μl/sample Protein A Dynabeads for 1 h. Then, DNA:RNA hybrids were washed by buffer A [20 mM Tris-HCl (pH = 8.0); 2 mM EDTA; 0.1% SDS; 1% Triton X-100; and 150 mM NaCl] once, buffer B [20 mM Tris-HCl (pH = 8.0); 2 mM EDTA; 0.1% SDS; 1% Triton X-100; and 500 mM NaCl] once, buffer C [10 mM Tris-HCl (pH = 8.0); 1 mM EDTA; 1% NP-40; 1% DOC; and 250 mM LiCl] once and buffer D [10 mM Tris-HCl (pH = 8.0) and 1 mM EDTA] twice. The elution of S9.6-DNA:RNA hybrids were achieved by rotating with 250 μl/sample elusion buffer (1% SDS and 100 mM NaHCO_3_) at room temperature for 30 min twice. DNA:RNA hybrids were then free by 2 h Proteinase K (55°C) incubation. The standard phenol/chloroform (Thermo Fisher, 10308293) process was used to extract DNA for the following qPCR. The detailed qPCR section is listed in the ChIP section.

### Chromatin-associated RNA sequencing/qPCR

The WT U2OS or *Asi*SI-ER U2OS cells were at 50–70% confluency in 15 cm dish (∼4–8 million cells). For DSB induction, 400 nM 4OHT was added to culture medium for 4 h. Cells were then washed and harvested into 5 ml PBS and pelleted by centrifuge (400 g, 5 min, 4°C). The cell pellets were lysed with 4 ml HLB + N buffer [10 mM Tris-HCl (pH = 7.5); 10 mM NaCl; 2.5 mM MgCl_2_; and 0.5% NP-40] and underlaid with 1 ml HLB + NS buffer [10 mM Tris-HCl (pH = 7.5); 10 mM NaCl; 2.5 mM MgCl_2_; 0.5% NP-40; and 10% sucrose] before being spinned at 400 g for 5 min (4°C) to collect the nuclear pellets. Cytoplasmic fragment was collected for western blot to confirm the successful breakage of cells. Next, nuclear pellets were lysed and resuspended in 125 μl NUN1 buffer [20 mM Tris-HCl (pH = 7.9); 75 mM NaCl; 0.5 mM EDTA; and 50% glycerol], and chromatin was extracted by incubating in 1.2 ml NUN2 buffer [20 mM HEPES-KOH (pH = 7.6); 300 mM NaCl; 0.2 mM EDTA; 7.5 mM MgCl_2_; 1% NP-40; and 1 M urea] on ice for 15 min with interval vortex in every 3–4 min. The chromatin samples were then collected by spinning at 13 000 rpm at 4°C for 15 min. To digest DNA and chromatin-associated proteins, 2 μl/sample Proteinase K (20 μg/μl)(NEB,P8107S) and 1 μl/sample Turbo DNase in 1× Turbo DNase Buffer (100 μl/sample) (Thermo fisher, AM2238) were added to digest chromatin pellets at 37°C with 1000 rpm shake until pellets were dissolved. RNA was extracted by trizol/chloroform RNA extraction, and further cleaning (to get rid of DNA contamination) with Monarch^®^ Total RNA Miniprep Kit (NEB, T2010S) by following manufacturer’s protocol. Sequencing library preparation was performed with the TruSeq Stranded Total RNA Sample Preparation Kit (Illumina) followed by paired-end sequencing on HiSeq2000 (Illumina). For chromatin-associated RNA (chrRNA)-qPCR, samples were subjected to qPCR (details in ChIP section) directly after trizol/chloroform RNA extraction.

### chrRNA sequencing data processing

chrRNA sequencing (chrRNA-seq) adapters were trimmed using Cutadapt (version 4.4) (https://cutadapt.readthedocs.io/en/stable/installation.html) in paired-end mode and the quality of the resulting fastq files were assessed using FastQC (https://www.bioinformatics.babraham.ac.uk/projects/fastqc/). The trimmed reads were then aligned to human hg19 reference genome using STAR aligner (51). Each alignment file was then split using Samtools into two alignment files containing positively stranded and negatively stranded reads (https://www.htslib.org/).

### Metagene plots

Strand specific coverage files containing Counts Per Million (CPM) normalized read count per nucleotide position was generated for each alignment using deepTools bamCoverage (https://deeptools.readthedocs.io/en/develop/) (52). ComputeMatrix operation of deepTools was then performed on the strand separated bigwig files to calculate the CPM coverage in the 2.5 kb flanking region of *Asi*SI site with bin size set at one. Bedtools intersect was used to find region of genes in 2.5 kb flank of each annotated *Asi*SI site. Then custom python script was employed to annotate the bin values in the positively stranded matrix as sense or antisense based on whether they lie in same or opposite orientation of gene regions near *Asi*SI respectively. Similarly, the negatively stranded matrix was annotated as sense or antisense using the above logic. Only bins laying within gene regions were utilized for sense/antisense annotation. The sense matrices from positively and negatively stranded matrices were concatenated to form a combined sense matrix containing read coverage in sense orientation around *Asi*SI site. Antisense matrix was also crafted in the same manner to represent antisense matrix in 2.5 kb flank region near each annotated *Asi*SI site. Antisense reads corresponding to different gene regions lying within same *Asi*SI site were summed to ensure that the matrix contains each *Asi*SI site as row with 5000 bins as columns representing antisense coverage in 2.5 kb flank region of annotated *Asi*SI. Similar procedure was also followed to generate sense matrix. Three *Asi*SI sites which do not overlap with any genes in 2.5 kb flanks were removed since the reads cannot be classified into sense and antisense. Matrix was then subdivided into different categories based on the known annotation of *Asi*SI sites as HR prone, NHEJ prone, uncut, highly transcriptionally active or transcriptionally less active sites. Sense and antisense matrix were then averaged across the *Asi*SI sites and plotted as line plots with separate scales using matplotlib python package. Fill plots representing read coverage in 2.5 kb flank region of individual *Asi*SI sites were also created using matplotlib as replacement for Integrative Genomics Viewer (IGV) snapshots.

### PCA plots

Bigwigsummary function of deeptools was employed in conjuction with Principal Component Analysis (PCA) function from scikit-learn to compare read coverage in 5 kb region of BLESS 80 *Asi*SI sites between sample replicates.

### Box plots

Coverage from sense and antisense matrices were used to make box plots representing CPM normalized reads in 500 bp flank region of each *Asi*SI site. Coverage was calculated by summing CPM values in 500 bins centered around DSB for each *Asi*SI site from sense and antisense matrix, respectively. Box plots were then made using matplotlib python package and significance determined with two-sample Wilcoxon test from scipy python package.

Fold changes across 500 bp region flanking *Asi*SI were calculated by taking ratio of coverage in these regions between condition and control matrix for sense and antisense separately. Log2 fold changes were then represented as box plots and significant difference in median values between sense and antisense were determined using two-sample Wilcoxon test.

### Heatmaps

plotHeatmap function of deepTools was used to make the heatmaps with regions set as all annotated DSBs arranged in ascending order of cleavage efficiency. Separate heatmaps were generated using the above procedure for sense and antisense matrices.

### ChIP-seq, DRIP-seq data processing

SETX + 4OHT ChIP-seq and S9.6 + 4OHT DNA:RNA hybrid immunoprecipitation (DRIP) samples were downloaded from Array Express (E-MTAB-6318). Read quality of the fastq files was checked using FastQC before and after adapter trimming. The trimmed reads were then mapped to hg19 genome following the standard ChIP-seq pipeline. The classic ChIP-seq pipeline consists of BWA (http://bio-bwa.sourceforge.net/) for alignment and samtools for duplicate removal (rmdup), sorting (sort) and indexing (index). Coverage files containing CPM normalized read count per nucleotide position was generated for each alignment file using deeptools bamCoverage (https://deeptools.readthedocs.io/en/develop/). ComputeMatrix operation of deepTools was then performed on the bigwig files to calculate the CPM coverage in the 2.5 kb flanking region of *Asi*SI site with bin size set to one.

### BLESS seq data processing

BLESS seq (E-MTAB-5817) was processed using the same protocol as detailed in (53). Read count coverage was calculated for all annotated DSBs (±500 bp) using bedtools multicov. The sites were then ordered based on read count coverage for representing cleavage efficiency of DSB sites.

### Fluorescence-activated cell sorting for reporter assay

The I-*Sce*I expression vector, pCBASceI plasmid (1.5 μg) (Addgene, 26477) (54), was transfected by using forward transfection with Lipofectamine 3000. After 48 h, cells were washed by ice-cold PBS twice and collected in 400 μl 10% FBS in PBS on ice before FACS. siBRCA1 was used as a control for HeLa HR reporter cells; the selective DNA-PK inhibitor BAY-8400 (Cambridge Bioscience, HY-132293–1mg) was used as a control for HeLa NHEJ reporter cells. Briefly, 2 μM BAY-8400 (BAY) was maintained in cell culture media until harvest. Sample data acquisition was achieved with CytoFLEX Flow cytometer (Beckman Coulter) and analyzed with FlowJo software.

### Clonogenic assay

A total of 1000 cells were seeded into a 12-well plate for 24 h before subjected to 0 and 2 Gy IR. The plate was then incubated at 37°C for 7–10 days until clear colonies formed. Colonies were fixed and stained with a 0.5% crystal violet (Sigma, C6158-100G) and 20% methanol (Merck, 32213–2.5L-M) for 1 h before washing by ddH_2_O. Plates was scanned and quantified by ImageJ with ColoneArea plugin.

### MTT assay

A total of 5000 cells (with siRNA knockdown) in 100 μl medium were seeded into a well of flat bottom 96‐well plates and culture for 24 h before subjected to 2 Gy IR. Then, cells were incubated for 24, 48 and 72 h, respectively. At corresponding time point, MTT cell proliferation assay kit (Abcam, ab211091) was used to measure cell viability by using the manufacturer’s protocol.

### Comet assay

A total of 5000 cells were fixed and embedded in 50 μl CometAssay LMAgarose (Bio-Techne, 4250–050-02) before being spotted on a Cometslide (Bio-Techne, 4250–050-03). At this point, the final concentration of low-melting gel is 0.5%. On-gel cell lysis was performed by placing the Cometslide into lysis buffer [2.5 M NaCl; 0.1 M EDTA; 10 mM Tris-base; 10% dimethylsulfoxide (DMSO) (freshly added); and 1% Triton X-100 (freshly added) (pH = 10)] at 4°C overnight. After rinsing with ddH_2_O, the Cometslide was immersed into running buffer [0.3 M NaOH, 1 mM EDTA, (pH = 13)] for 1 h at 4°C before electrophoresed at constant 300 mA for 0.5 h. The neutralization step was performed with 0.4 M Tris-base buffer (pH = 7.5) for 5 min at room temperature twice before the Cometslide been washed by 70% ethanol for 15 min and air dried. The chromatin was stained with 2 μg/ml DAPI (BD Biosciences, 564907) in PBS for 5 min followed by 5 min ddH_2_O washing. Images was acquired with EVOS M7000 microscope with 10× objectives. ImageJ with OpenComet plugin was used for the tail moment quantification. The significance was determined by using unpaired Welch’s correction.

Details about all key reagents used in this study can be found in Supplementary Table S5.

Details about sequences of oligonucleotides used in this study can be found in Supplementary Tables S1–S3. The list of cut *Asi*SI sites used for metagene plots and further downstream analysis are listed in Supplementary Table S4.

### Statistical analysis

Statistical tests were performed in GraphPad Prism 10.3.1. All error bars represent mean ±SD unless stated differently. Each experiment repeats at least three times (N = 3). The Kolmogorov–Smirnov normality test was performed to test for a normal distribution. If data meets normal distribution, statistical testing was performed using the Student's *t*-test, one-way ANOVA (Analysis of Variance), or unpaired Welch’s correction (for comet assay analysis). If data did not show a normal distribution, Mann–Whitney test for two groups (non-parametric comparison for PLA foci analysis), or Dunn’s test with Bonferroni corrections for multiple group comparisons were performed. Significances are listed as *P ≤ 0.05, **P ≤ 0.01, ***P≤ 0.001, ****P≤ 0.0001.

## Results

### INTS6 forms a tetrameric SOSS1 complex and binds to RNAPII in response to DNA damage

Previous research has demonstrated the *in vitro* formation of a tetrameric complex, involving Integrator complex subunit 6 (INTS6) and the trimeric SOSS1 complex, comprised of INTS3, hSSB1/2 and INIP (40–42). Utilizing PLA, we observed increased proximity between INTS6 and INTS3, hSSB1 or INIP following ionizing radiation (IR) (Supplementary Figure S1A–C). These data were further confirmed by co-immunoprecipitation (co-IP) (Supplementary Figure S1D).

Previously, we have shown that the trimeric SOSS1 complex interacts with RNAPII in response to DNA damage (38). Therefore, we tested whether the tetrameric SOSS1 complex plays a role in the regulation of RNAPII transcription. First, we used the irreversible RNAPII inhibitor TPL in combination with PLA and observed a transcription-dependent recruitment of INTS3 and INTS6, respectively, to γH2AX upon IR treatment (Supplementary Figure S2A and B). Furthermore, we confirmed transcription-dependent recruitment of INTS6 to DSBs by laser microirradiation (Supplementary Figure S2C).

Next, we explored the proximity of INTS6 to total RNAPII and active RNAPII phosphorylated at Ser2 (S2P), Ser5 (S5P) and Tyr1 (Y1P), respectively. We identified increased proximity between INTS6 and all forms of RNAPII tested following IR (Supplementary Figure S3A). These data were further supported by co-IP showing increased interaction between INTS6 and RNAPII variants upon IR (Supplementary Figure S3B–D).

Furthermore, treatment of cells with transcription inhibitors such as DRB (inhibitor of CDK9, phosphorylating Ser2 on the CTD) and THZ1 (inhibitor of CDK7, phosphorylating Ser5 on the CTD) significantly reduced the number of PLA foci between INTS6 and S2P- or S5P-RNAPII (Supplementary Figure S3E and F). It should be noted that RNAPII has 52 heptapeptides, and would not be exclusively phosphorylated on S2 or S5. Therefore, even with a specific inhibitor, we could observe the reduced proximity between both S2P or S5P and INTS6.

To test whether INTS6 interacts directly with RNAPII, we conducted *in vitro* pull-down experiments using purified INTS6 and the tetrameric SOSS1 complex (Supplementary Figure S4A and B), as well as unphosphorylated GST-CTD, Y1P CTD and S5,7P CTD, respectively. Our data indicate that all three tested variants of GST-CTD polypeptides pulled down the tetrameric SOSS1 complex, but not the isolated INTS6 alone, suggesting that formation of the complex is required for the direct interaction with RNAPII CTD (Supplementary Figure S4C and D). To provide a quantitative understanding of the binding between SOSS1 complexes and CTD polypeptides, we used MST analysis. The results revealed that both trimeric and tetrameric SOSS1 complexes exhibited similar affinities for unphosphorylated CTD and Y1P CTD (Supplementary Figure S4E and F). However, the tetrameric SOSS1 complex displayed higher affinity for S5,7P CTD (Supplementary Figure S4G) compared to the trimeric SOSS1 complex.

### INTS6 localizes to DSBs in a DNA:RNA hybrid-dependent manner

**Figure 1.**
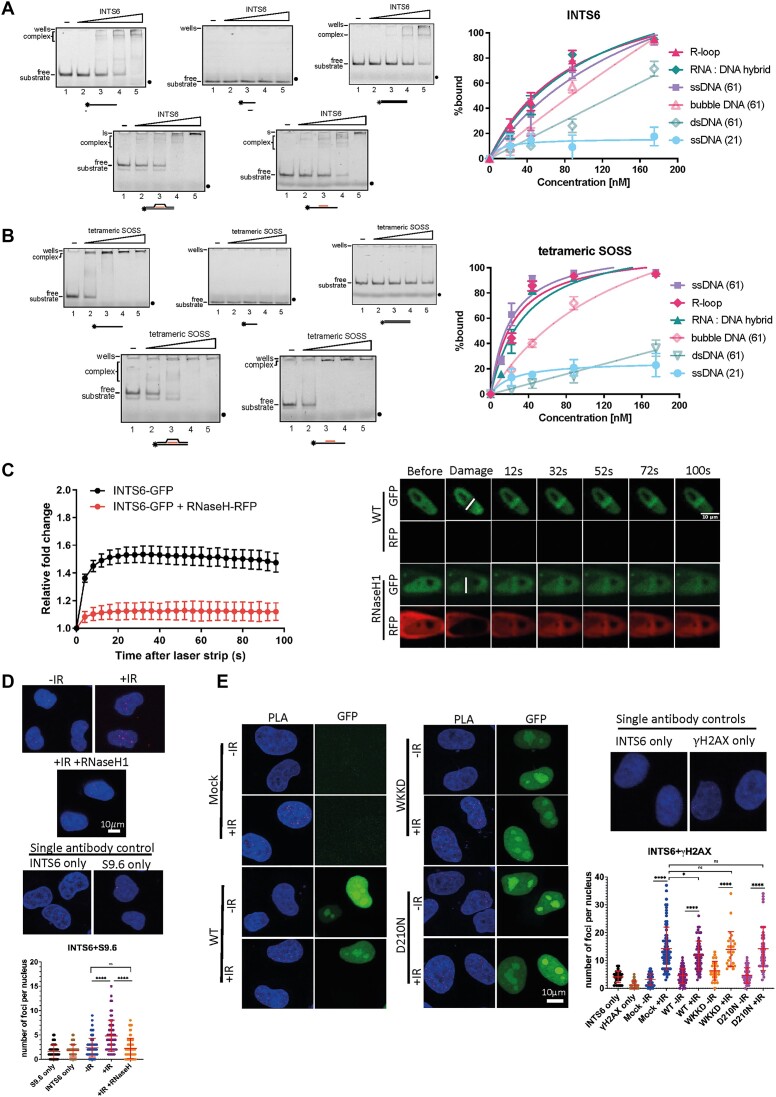
INTS6 localizes to DSBs in a DNA:RNA hybrid-dependent manner. (**A**) Left: Scans of representative EMSA experiments of INTS6 with 61- or 21-mer ssDNA, 61-mer dsDNA, R-loops and DNA:RNA hybrids. Right: Graph representing quantification of EMSA experiments (n = 3). (**B**) Left: Scans of representative EMSA experiments of the tetrameric SOSS1 complex with 61- or 21-mer ssDNA, 61-mer dsDNA, R-loops and DNA:RNA hybrids. Right: Graph representing quantification of EMSA experiments (n = 3). (**C**) Laser striping of cells transiently transfected with INTS6-GFP plasmid with or without RNase H1-RFP plasmid. White lines indicate laser stripes. Representative confocal microscopy images and quantification (n ≥ 20) show GFP and RFP signals at the indicated time points. Error bars: mean ± SEM. (**D**) PLA of INTS6 and S9.6 in cells without IR, with IR or with IR and RNaseH1 treatment. IR = 10 Gy, samples were collected 10 min post-IR. Here, we used modified PLA protocol (55), which includes RNaseT1 (digest ssRNA) and RNase III (digest dsRNA) treatment during slide preparation process. Top: Representative confocal microscopy images; bottom: quantification of top, error bar = mean ± SD, significance was determined using non-parametric Mann–Whitney test. ****P ≤ 0.0001. Scale bar = 10 μm. Single antibodies were used as a negative control. (**E**) PLA of INTS6 and γH2AX in cells transiently transfected with RNAseH1^wt^-GFP or RNAseH1^WKKD^-GFP (binding and catalytic) or RNAseH1^D210N−^GFP (catalytic) mutants with or without IR. IR = 10 Gy, samples were collected 10 min post-IR. Left: representative confocal microscopy images; right: quantification of left, error bar = mean ± SD, significance was determined using non-parametric Mann–Whitney test. ****P ≤ 0.0001, *P ≤ 0.05. Scale bar = 10 μm. Single antibodies were used as a negative control.

A recent study has shown that the SOSS1–Integrator–PP2A complex binds to R-loops at the promoters of protein-coding genes through the hSSB1 subunit in non-damage conditions (43). Our previous work demonstrated that the trimeric SOSS1 complex, specifically through its hSSB1 subunit, binds to various nucleic acids (NA) structures, including ssDNA, DNA:RNA hybrids and R-loops, whilst the INTS3 subunit alone did not exhibit binding to these NA substrates (38). INTS6, as a putative DEAD box helicase, may bind NAs, thereby modulating the binding pattern of the tetrameric SOSS1 complex. In this study, we utilized *in vitro* electrophoretic mobility shift assay (EMSA) to assess the binding properties of INTS6 to various NA substrates, including a 61-mer ssDNA, 21-mer ssDNA, 61-mer double-stranded DNA (dsDNA), DNA:RNA hybrids and R-loops. Our findings indicate that INTS6 binds to DNA:RNA hybrids and R-loops with the highest affinity. Moreover, it binds to ssDNA in a length-dependent manner (Figure 1A). The tetrameric SOSS1 complex exhibited a similar binding pattern to INTS6 alone, albeit with different affinities (the tetrameric complex exhibiting higher affinity), stemming from the presence of a second DNA binding protein – hSSB1 (Figure 1B), suggesting that the inclusion of INTS6 into the tetrameric SOSS1 does not alter its NA binding properties, when compared with the trimeric SOSS1 complex. It should be noted that these EMSA experiments are complemented by our previously published work and show the binding of the other constituents of the trimeric SOSS1 complex to NA. In those experiments, we showed that His-tagged INTS3 did not bind nucleic acid substrates, suggesting that the tags alone play no role in the observed nucleic acid binding by INTS6 and the tetrameric SOSS1 (38).

To test whether INTS6 recruitment to DSBs is dependent on DNA:RNA hybrids, we performed laser microirradiation in cells expressing INTS6-GFP and RNAseH1-RFP. We detected a rapid recruitment of INTS6 to DSBs in WT cells, which was reduced by the overexpression of RNAseH1 (Figure 1C). To further investigate whether INTS6 is in close proximity to DNA:RNA hybrids, we performed a modified PLA using antibodies against INTS6 and S9.6 (recognizing DNA:RNA hybrids) (55) and observed a significant increase in PLA foci following IR (Figure 1D).

Subsequently, we transfected cells with plasmids expressing RNAseH1^WT^-GFP (known to resolve DNA:RNA hybrids), RNAseH1^D210N^-GFP (a catalytically inactive mutant) and RNAseH1^WKKD^-GFP (a combined binding and catalytic mutant) (Supplementary Figure S4H). We then conducted PLA with antibodies against INTS6 and γH2AX. Our results revealed a significant increase in PLA foci in mock cells and cells expressing RNAseH1^D210N^-GFP or RNAseH1^WKKD^-GFP, but not in those expressing RNAseH1^WT^-GFP (Figure 1E).

Overall, these data suggest that INTS6 is recruited to DSBs in a DNA:RNA hybrid-dependent manner.

### INTS6 stimulates PP2A recruitment to DSBs

Integrator–PP2A complex plays a crucial role in dephosphorylating RNAPII at S2 and S5 of the CTD in non-damage conditions. The phosphatase module formed by PP2A includes INTS6, which aids in the assembly of PP2A into the Integrator–PP2A complex (36). Additionally, PP2A has been identified to bind BRCA2 and to promote HR (56). Despite these known functions, the role of PP2A in transcription regulation at DSBs remains elusive.

**Figure 2.**
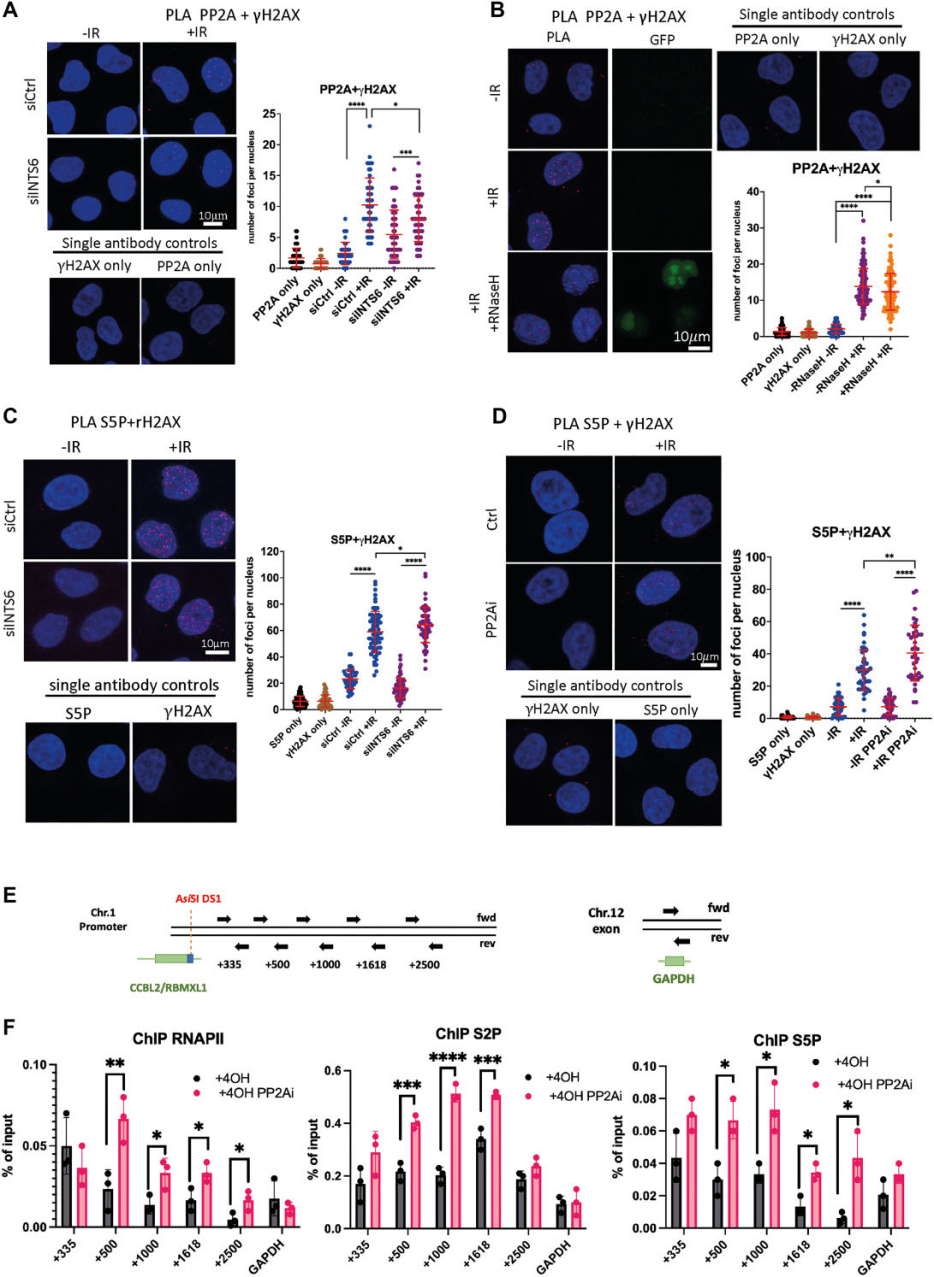
INTS6 facilitates PP2A recruitment to DSBs to dephosphorylate RNAPII. (**A**) PLA of PP2A and γH2AX in WT or INTS6 knockdown cells with or without IR. IR = 10 Gy, samples were collected 10 min post-IR. Left: Representative confocal microscopy images; right: quantification of left, error bar = mean ± SD, significance was determined using non-parametric Mann–Whitney test., ****P ≤ 0.0001, ***P ≤ 0.001, *P ≤ 0.05. Scale bar = 10 μm. Single antibodies were used as a negative control. (**B**) PLA of PP2A and γH2AX in cells with or without transient RNaseH1-GFP expression, with or without IR. IR = 10Gy, samples were collected 10 min post-IR. Left: Representative confocal microscopy images; right: quantification of left, error bar = mean ± SD, significance was determined using non-parametric Mann–Whitney test. ****P ≤ 0.0001, *P ≤ 0.05. Scale bar = 10 μm. Single antibodies were used as a negative control. (**C**) PLA of S5P and γH2AX in wildtype or INTS6 knockdown cells with or without IR. IR = 10 Gy, samples were collected 10 min post-IR. Left: Representative confocal microscopy images; right: quantification of left, error bar = mean ± SD, significance was determined using non-parametric Mann–Whitney test. ****P ≤ 0.0001, *P ≤ 0.05. Scale bar = 10 μm. Single antibodies were used as a negative control. (**D**) PLA of S5P and γH2AX with or without IR in the presence or absence of PP2A inhibitor (LB-100, 2.5 μM, 2 h). IR = 10 Gy, samples were collected 10 min post-IR. Left: Representative confocal microscopy images; right: quantification of left, error bar = mean ± SD, significance was determined using non-parametric Mann–Whitney test. ****P ≤ 0.0001, **P ≤ 0.01. Scale bar = 10 μm. Single antibodies were used as a negative control. (**E**) Left: Drawing of ChIP probes positions around DS1. Right: drawing showing position of ChIP probes in GAPDH gene. (**F**) Bar charts showing RNAPII, S2P and S5P ChIP signals at DS1 in the absence or presence of PP2A inhibitor (LB-100, 2.5 μM, 4 h). n ≥ 3. Error bar = mean ± SD, significance was determined using unpaired Student’s *t*-test, ****P ≤ 0.0001, ***P ≤ 0.001,**P ≤ 0.01*P ≤ 0.05.

To investigate whether the recruitment of PP2A to DSBs is dependent on INTS6, we employed PLA using antibodies against PP2A and γH2AX in control and INTS6 depleted cells in the presence or absence of IR. Our results revealed a significant increase in the interaction between PP2A and γH2AX upon IR, which was reduced in the absence of INTS6, indicating that INTS6 stimulates the recruitment of PP2A to DSBs (Figure 2A). To investigate DSBs in a sequence-specific manner, we utilized the U2OS-A*si*SI-ER cell line, in which the A*si*SI restriction enzyme is fused to the oestrogen receptor ligand-binding domain. Addition of 4-OHT induces translocation of the A*si*SI-ER enzyme to the nucleus where it recognizes 5′-GCGATCGC-3′ sequence motifs and generates site-specific cuts simulating DSBs at specific genomic loci (57,58). There are 1231 predicted A*si*SI-ER cleavage sites in the human genome, but only 80 sites are efficiently cut *in vivo*, as it has been validated by γH2AX occupancy (13,57). Using this system, we showed that the Y1P-modified RNAPII transcribes nascent RNAs (DARTs) (12). We selected two DSBs, DS1 (A*si*SI cut site in the promoter region of CCBL2/RBMXL1 gene on chromosome 1) and DS2 (in the intron 3 region of SEEK1/PSORS1C1 gene on chromosome 6) for further experiments (Supplementary Figure S5A) and confirmed successful, site-specific cuts by ChIP using a γH2AX antibody (Supplementary Figure S5B). Using ChIP, we detected PP2A at two selected DSBs and this was reduced in cells depleted of INTS6 (Supplementary Figure S5C). We have shown above that INTS6 is recruited to DSBs in a DNA:RNA hybrid-dependent manner in Figure 1. Therefore, we tested whether a reduction in DNA:RNA hybrid levels could consequently affect PP2A levels at DSBs. Indeed, PLA revealed that the overexpression of RNAseH1 reduced PP2A levels at DSBs (Figure 2B). Furthermore, these data were confirmed by ChIP at two selected DSBs (Supplementary Figure S5D).

Next, we explored the interaction between PP2A and RNAPII and detected an increased number of PLA foci in response to DNA damage. Importantly, these PLA foci were significantly reduced upon INTS6 knockdown, emphasizing the crucial role of INTS6 in facilitating the proximity of PP2A to RNAPII (Supplementary Figure S6A). Consequently, we also tested whether reduced levels of DNA:RNA hybrids would affect PP2A proximity to RNAPII upon IR. Indeed, PLA revealed that the overexpression of RNAseH1 reduced the number of PP2A/RNAPII foci (Supplementary Figure S6B).

In summary, these data suggest that INTS6 plays a role in facilitating the recruitment of PP2A to DSBs in a R-loop-dependent manner.

### PP2A is required for the dephosphorylation of RNAPII at DSBs

Next, we sought to determine whether PP2A plays a role in the dephosphorylation of RNAPII at DSBs. Initially, we observed an increased occupancy of active RNAPII phosphorylated at S5 at DSBs in cells depleted of INTS6, indicating impaired RNAPII dephosphorylation in the absence of INTS6. This defect may be attributed to the absence of PP2A at DSBs (Figure 2C). To further support this observation, we examined the occupancy of total RNAPII and S5P RNAPII by ChIP at two selected DSBs and observed increased levels of RNAPII and S5P RNAPII in cells depleted of INTS6 (Supplementary Figure S6C and D).

Next, we investigated RNAPII occupancy at DSBs following the treatment with PP2A inhibitor, LB-100. We detected significantly enriched PLA foci corresponding to proximity between RNAPII, S5P RNAPII and γH2AX upon IR, and this effect was further exacerbated when PP2A was inhibited (Figure 2D and Supplementary Figure S7A). Subsequent ChIP experiments using RNAPII-S2P and -S5P RNAPII-specific antibodies revealed increased levels of RNAPII at DS1 and DS2 upon PP2A inhibition. The levels of RNAPII were not affected at the GAPDH locus, used as a negative control (Figure 2E, F and Supplementary Figures S5A and S7B). The increased levels of RNAPII at DSBs following PP2A inhibition indicate that PP2A may be required for the dephosphorylation of RNAPII at DSBs.

Interestingly, PP2A inhibition resulted in a significant increase in the number of PLA foci corresponding to the proximity of INTS6 to γH2AX (Supplementary Figure S7C). This was further confirmed by laser microirradiation, showing increased INTS6-GFP accumulation at DSBs in cells treated with PP2A inhibitors (Supplementary Figure S7D). As established earlier, INTS6 binds to phosphorylated RNAPII, and the increased number of INTS6/γH2AX PLA foci upon PP2A inhibition may reflect increased levels of S5P RNAPII at DSBs.

### INTS6 depletion leads to the accumulation of DARTs

**Figure 3.**
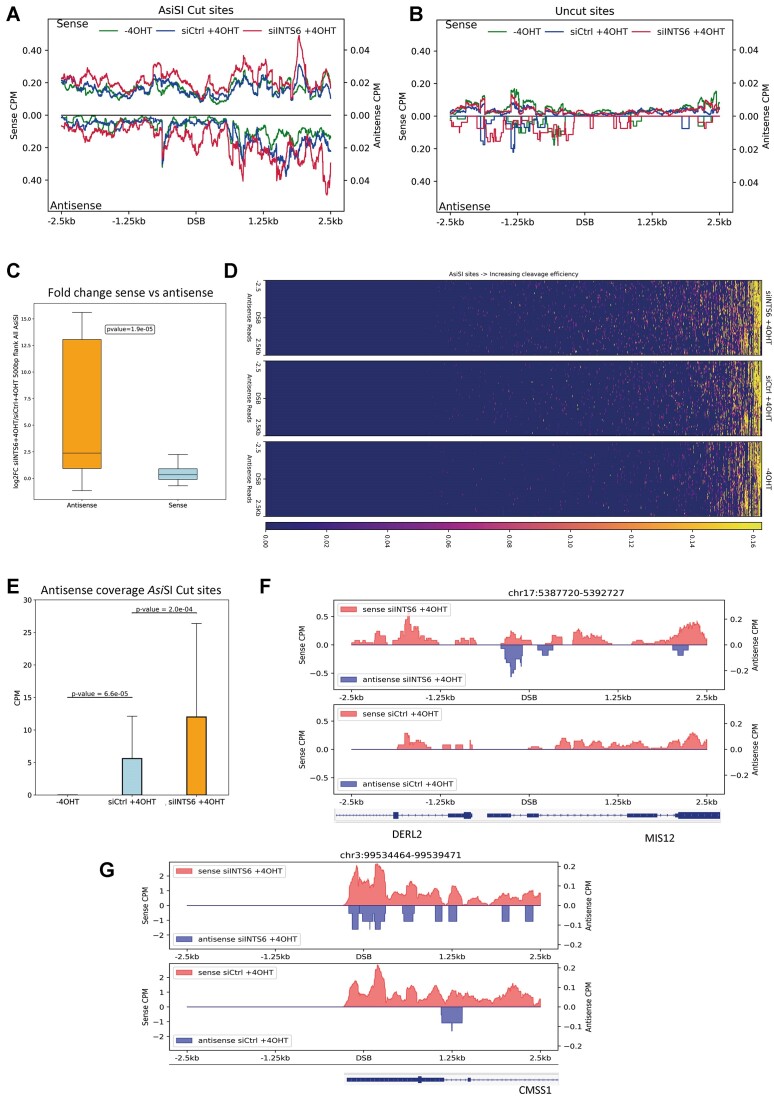
INTS6 depletion leads to the accumulation of DARTs. (**A**) Metagene plot shows chrRNA-seq sense and antisense coverage in no damage (-4OHT), damage (siCtrl + 4OHT) and INTS6 knockdown (siINTS6 + 4OHT) cells around 2.5 kb flank region of cut *Asi*SI sites (details in Supplementary Table S4). The reference genome is human hg19. (**B**) Metagene plot shows chrRNA-seq sense and antisense coverage in no damage (-4OHT), damage (siCtrl + 4OHT) and INTS6 knockdown (siINTS6 + 4OHT) cells around 2.5 kb flank region of uncut *Asi*SI sites (details in Supplementary Table S4). The reference genome is human hg19. (**C**) Box plots show log2 fold change of chrRNA-seq coverage of sense reads and antisense reads upon INTS6 knockdown with damage induction compared to control with damage induction for cut *Asi*SI (± 500 bp). Two-sample Wilcoxon test is used for statistical testing of medians between sense and antisense log2 fold change distribution. (**D**) Heatmaps show antisense nascent RNA (chrRNA-seq) read coverage across annotated *Asi*SI sites (details in Supplementary Table S4) sorted based on their cleavage efficiency. (**E**) Box plot shows chrRNA-seq antisense coverage in ± 500 bp flank of *Asi*SI cut sites (details in Supplementary Table S4) in -4OHT, siCtrl + 4OHT and siINTS6 + 4OHT samples. Two-sample Wilcoxon test is used with medians test. Error bar = mean ± SD. (**F, G**) Representative snapshots of individual genes showing sense and antisense chrRNA-seq coverage in INTS6 knockdown and control with damage induction around 2.5 kb flank region of *Asi*SI cut. The specific loci information is listed on top of the snapshots. The reference genome is human hg19.

To investigate the impact of the accumulation of phosphorylated RNAPII in cells depleted of INTS6 on a genome-wide scale, we conducted chrRNA-seq in U2OS-*Asi*SI-ER cells. Chromatin-associated RNAs were isolated from both control (siCtrl, scrambled siRNA) and INTS6-depleted cells (siINTS6) in the presence or absence of 4-OHT and subjected to next-generation sequencing. Principal component analysis revealed the clustering of sample replicates, showing robust data reproducibility (Supplementary Figure S8A). The analysis of chrRNA-seq data and RT-qPCR revealed only a marginal increase in DARTs following induction of damage by addition of 4OHT (Figure 3A and Supplementary Figure S8B). We hypothesized that DARTs are rapidly processed, therefore are very hard to detect. Indeed, we observed a significant increase in the nascent transcript levels surrounding DSBs [cut *Asi*SI sites (53)] in the INTS6-knockdown cells, compared to the control samples (Figure 3A and Supplementary Figure S8B). In contrast, uncut sites (used as a negative control) exhibited consistently low levels of the nascent transcripts (Figure 3B). Next, we categorized the nascent RNAs based on their direction relative to the transcriptional direction of genes at the *Asi*SI cuts, labelling them as sense (transcribed in the same direction as the nearby gene) or antisense (transcribed in the opposite direction as the nearby gene). Intriguingly, the depletion of INTS6 resulted in significantly increased levels of nascent RNAs in both directions, with a more pronounced increase in the antisense RNA levels (Figure 3A, C, D and E, and Supplementary Figure S8C). This observation was anticipated, as a certain amount of sense RNA is pre-synthesized before DSBs induction, whereas antisense RNA represents *de novo* transcription (Figure 3F and G).

*Asi*SI cut sites can be characterized based on their proximity to highly transcribed regions (53). The analysis of chrRNA-seq in the INTS6-depleted cells indicated a significant increase in both the sense and the antisense RNA transcripts around DSBs in proximity to highly transcribed genes. However, a significant increase was observed only in the sense RNA transcripts near genes with low transcription activity (Supplementary Figure S9A–D). This supports the previous findings That pre-existing transcriptional states may influence DNA repair (59). Previous studies have shown that transcription and R-loops may act as a recruitment platform for HR repair factors (13,38). The *Asi*SI cut sites may be classified into HR or NHEJ prone DSBs, based on the correlation ratio using ChIP-seq data coverage of RAD51, a key HR factor, (*Asi*SI site ± 4 kb) and XRCC4, a known NHEJ factor, coverage (*Asi*SI site ± 1 kb). The top 30 sites were annotated as HR prone, due to a higher RAD51 coverage, and the bottom 30 as NHEJ prone, due to a higher ratio of XRCC4 (53). chrRNA-seq analysis revealed a significant increase in nascent transcripts in INTS6-depleted cells for DSBs repaired by both pathways (Supplementary Figure S9E–H).

In conclusion, our data suggest that the depletion of INTS6 leads to elevated levels of nascent RNA around DSBs. This could imply that INTS6 facilitates the processing of DARTs.

### INTS6 interacts with SETX

**Figure 4.**
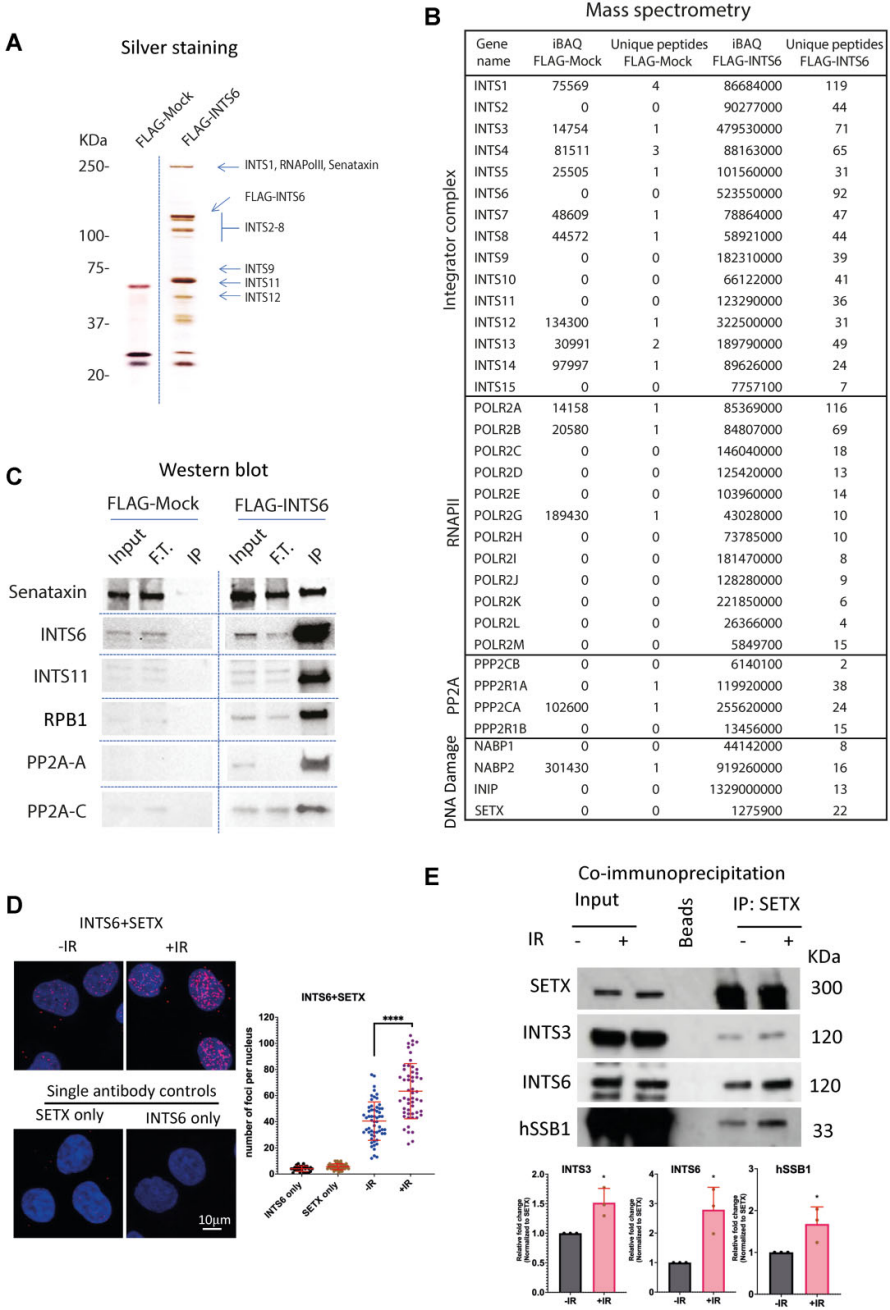
INTS6 associates with SETX. (**A**) Silver stain of affinity-purified Integrator complex. The integrator complex was purified from nuclear lysate of HEK293Tcells, stably overexpressing FLAG-INTS6. Mock indicates the same FLAG-IP purification steps from parental HEK293T cells. The indicated Integrator subunits were assigned as identified by Baillat *et al.* (31). (**B**) Affinity-purified Integrator complex mass spectrometry analyses were performed on nuclear lysate of HEK293T cells stably overexpressing FLAG-INTS6 or mock FLAG-IP purification steps from parental HEK293T cells. The values represent intensity-based absolute quantification (iBAQ) intensities and the unique peptides. (**C**) Affinity-purified Integrator complex followed by western blot showing indicated proteins. (**D**) PLA of SETX and INTS6 with or without IR treatment. IR = 10 Gy, samples were collected 10 min post-IR. Left: Representative confocal microscopy images; right: quantification of left, error bar = mean ± SD, significance was determined using non-parametric Mann–Whitney test. ****P ≤ 0.0001. Scale bar = 10 μm. Single antibodies were used as a negative control. (**E**) Immunoprecipitation of SETX from cells with or without IR treatment (IR = 10 Gy, samples were collected 10 min post-IR), followed by Western blot showing signals for SETX, INTS6, INTS3 and hSSB1. KDa indicates size of the proteins. Bar charts show quantifications of three independent blots, error bar = mean ± SD, significance was determined using unpaired Student’s *t*-test, *P ≤ 0.05.

The accumulation of DARTs in cells depleted of INTS6 suggests that INTS6, either directly or indirectly, plays a role in their processing. To identify proteins interacting with INTS6, we conducted affinity purification of nuclear FLAG-INTS6. Silver staining of mock and FLAG-INTS6 pull-downs indicated the presence of INTS6-specific bands, potentially corresponding to other Integrator subunits based on their molecular weight (Figure 4A). Indeed, mass spectrometry analysis of FLAG-INTS6 pull-downs confirmed the presence of all 15 subunits of the Integrator complex. Additionally, we identified subunits of RNAPII, PP2A kinase and SOSS complexes, including NABP1 (hSSB2), NAPBP2 (hSSB1) and INIP, in the INTS6 pull down samples. Notably, we also recovered SETX as an INTS6-associated polypeptide (Figure 4B). SETX, was described as an RNA/DNA helicase involved in R-loop resolution, transcription termination (44,45), RNA splicing, RNA processing, RNA stability (60) and coordination of replication and transcription conflicts (61). Additionally, SETX was recently identified to function as a *bona fide* transcription termination factor (45). We validated the findings from mass spectrometry by analysing the affinity-purified eluates from FLAG mock and FLAG-INTS6 IPs using SETX, INTS11, RBP1, PP2A-A and PP2A-C specific antibodies. Specific signals for all tested proteins were observed in FLAG-INTS6 samples, but not in FLAG mock samples (Figure 4C).

Subsequently, we investigated whether the INTS6-SETX interaction could be detected under DNA damage conditions. PLA revealed INTS6 in close proximity to SETX in non-damage conditions and this interaction was further enhanced by DNA damage (Figure 4D). Finally, we repeated the co-IP using antibodies against endogenous SETX in cells exposed to IR. Western blot analysis confirmed the interaction between SETX and INTS6, with an augmentation of this interaction in DNA damage conditions (Figure 4E). Furthermore, we also detected INTS3 and hSSB1 in SETX pull-down following IR, suggesting that DNA damage stimulates interaction between SETX and tetrameric SOSS1 complex. Depletion of INTS6 reduced the interaction between SETX and INTS3, suggesting that the proximity between SETX and SOSS1 is mediated by INTS6 (Supplementary Figure S10A).

In summary, these results revealed a previously unreported interaction between tetrameric SOSS1 complex and SETX, which is stimulated by DNA damage.

### INTS6 is required for SETX recruitment and resolution of DNA:RNA hybrids at DSBs

**Figure 5.**
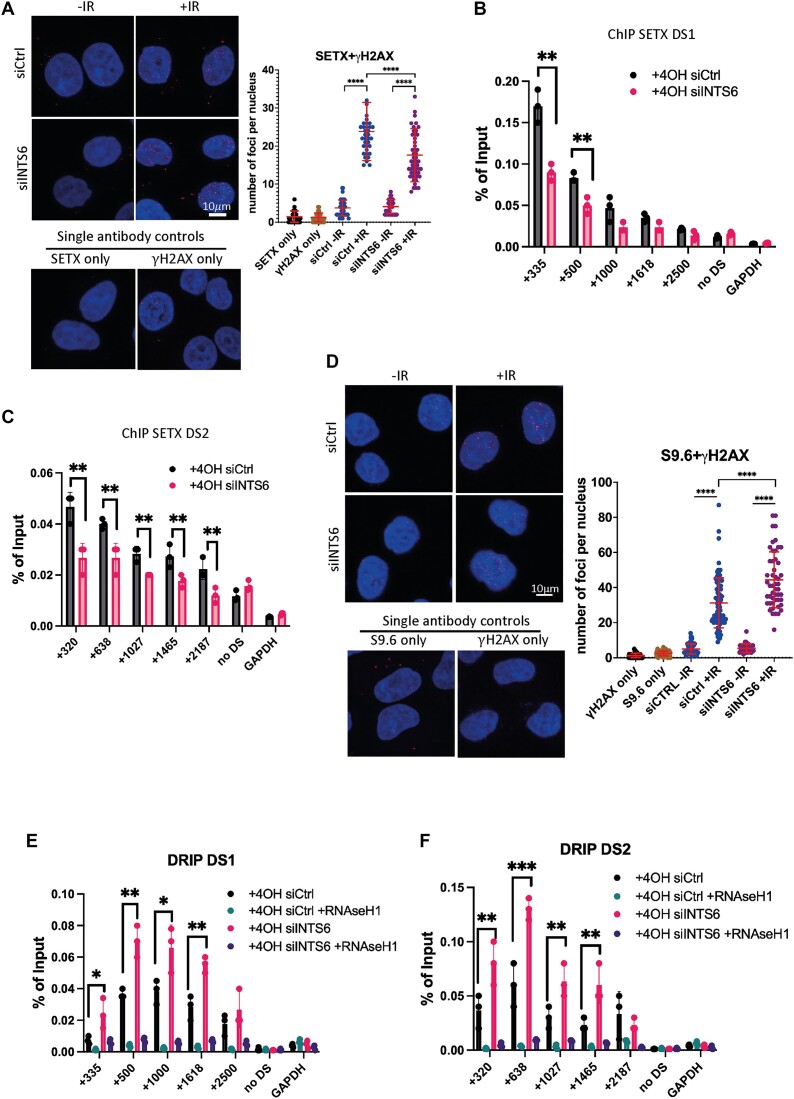
INTS6 is required for SETX recruitment to DSBs and clearance of DNA:RNA hybrids. (**A**) PLA of SETX and γH2AX with or without IR in mock or siINTS6 cells. IR = 10 Gy, samples were collected 10 min post-IR. Left: Representative confocal microscopy images; right: quantification of left, error bar = mean ± SD, significance was determined using non-parametric Mann–Whitney test. ****P ≤ 0.0001. Scale bar = 10 μm. Single antibodies were used as a negative control. (**B, C**) Bar charts showing SETX ChIP signals over DS1 (**B**) and DS2 (**C**) loci in the presence or absence of INTS6. n = 3. Error bar = mean ± SD, significance was determined using Student’s *t*-test, unpaired, **P ≤ 0.01. (**D**) PLA of S9.6 and γH2AX with or without IR in mock or siINTS6 cells. IR = 10 Gy, samples were collected 10 min post-IR. Left: Representative confocal microscopy images; right: quantification of top, error bar = mean ± SD, significance was determined using non-parametric Mann–Whitney test. ****P ≤ 0.0001. Scale bar = 10 μm. Single antibodies were used as a negative control. (**E, F**) Bar charts showing DRIP signals over DS1 (**E**) and DS2 (**F**) loci in the presence or absence of INTS6 and RNaseH1 treatment. n = 3. Error bar = mean ± SD, significance was determined using unpaired Student’s *t*-test, ***P ≤ 0.001, **P ≤ 0.01, *P ≤ 0.05.

To investigate the functionality of the interaction between INTS6 and SETX, we explored the potential requirement of INTS6 for the recruitment of SETX to DSBs. PLA confirmed the proximity of SETX to γH2AX, which was INTS6 dependent (Figure 5A). We further investigated the presence of SETX at specific DSBs by ChIP and observed significantly reduced levels of SETX at two selected DSBs in INTS6 knockdown samples. SETX was not detected at the control site (no DSB, chr22:23141639–23141780) nor at the GAPDH locus (Figure 5B and C). These results suggest an INTS6-dependent recruitment of SETX to the sites of DNA damage.

To investigate whether SETX modulates levels of DNA:RNA hybrids in an INTS6-dependent manner upon DNA damage, we employed PLA using S9.6 and γH2AX antibodies in WT and INTS6-depleted cells treated or not with IR. We detected a significant increase in the DNA:RNA hybrid levels upon IR, which was further exacerbated by INTS6 depletion (Figure 5D). DRIP analysis, using the S9.6 antibody to capture DNA:RNA hybrid levels around DSBs, revealed increased levels of the hybrids in cells depleted of INTS6 at two selected DSBs. Levels of DNA:RNA hybrids at control loci (no DSB and GAPDH) remained unchanged (Figure 5E and F).

These findings suggest that the absence of INTS6 impairs the recruitment of SETX to DSBs, resulting in prolonged existence of DNA:RNA hybrids. This underscores the importance of INTS6 in maintaining the homeostasis of DNA:RNA hybrids at DSBs.

### INTS6-dependent accumulation of DARTs correlates with DNA:RNA hybrids at DSBs

**Figure 6.**
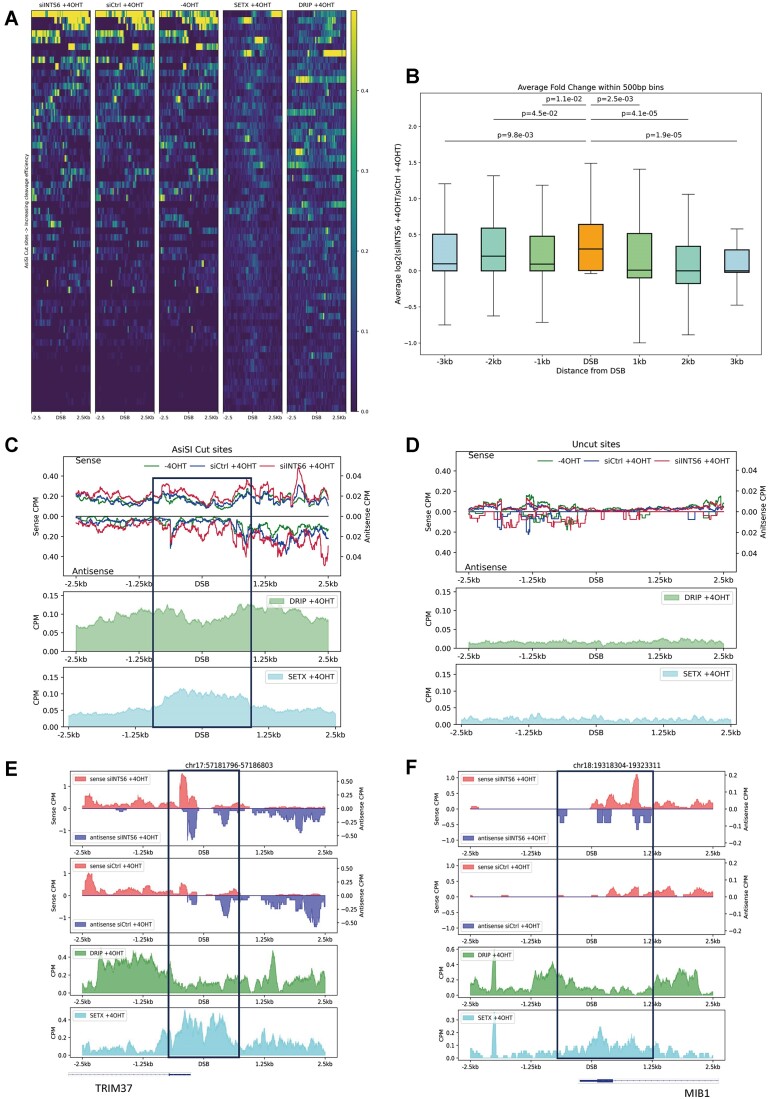
INTS6-dependent accumulation of DARTs correlates with DNA:RNA hybrids at DSBs. (**A**) Heatmaps show the chrRNA-seq coverage in siINTS6 + 4OHT, siCtrl + 4OHT, -4OHT, SETX + 4OHT ChIP-seq coverage and S9.6 + 4OHT DRIP-seq coverage across cut *Asi*SI sites (details in Supplementary Table S4) sorted by their cleavage efficiency. The reference genome is human hg19. (**B**) Box plots show log2 fold change of total chrRNA-seq coverage of INTS6 knockdown with damage induction compared to control with damage induction in 500 bp bins centered at DSB, 1, 2 and 3 kb away from DSB (cut *Asi*SI sites, details in Supplementary Table S4). The reference genome is human hg19. (**C**) Metagene plots show S9.6 DRIP-seq coverage and SETX coverage upon damage induction along with chrRNA-seq sense and antisense coverage around 2.5 kb flank region of cut *Asi*SI sites (details in Supplementary Table S4). The reference genome is human hg19. (**D**) Metagene plots show S9.6 DRIP-seq coverage and SETX coverage upon damage induction along with chrRNA-seq sense and antisense coverage around 2.5 kb flank region of uncut *Asi*SI sites (n = 20). The reference genome is human hg19. (**E, F**) Representative snapshots of individual genes showing DRIP-seq coverage and SETX coverage upon damage induction along with sense and antisense chrRNA-seq coverage in siINTS6 and control with damage induction around 2.5 kb flank region of *Asi*SI cut. The specific loci information is listed on top of the snapshot respectively. The reference genome is human hg19.

DARTs and dilncRNA can form DNA:RNA hybrids and R-loops at DSBs by hybridizing to the DNA overhangs after resection or unwound DNA behind paused RNAPII (12,17). Analyses of SETX ChIP-seq and S9.6 DRIP-seq in U2OS-A*si*SI-ER cells have indicated that SETX localizes to DSBs, playing a critical role in resolving DNA:RNA hybrids and safeguarding genome stability (22). In this study, we integrated our chrRNA-seq datasets with SETX ChIP-seq and S9.6 DRIP-seq datasets to examine whether INTS6-dependent DARTs correlate with SETX and DNA:RNA hybrid occupancy around DSBs. Heatmaps displaying the levels of nascent RNA from control and siINTS6 chrRNA-seq data, in conjunction with SETX ChIP-seq and S9.6 DRIP-seq datasets, revealed that DSBs with high cleavage efficiency tend to have more RNA transcripts, more SETX and more DRIP signals than DSBs with low cleavage efficiency (Figure 6A and Supplementary Figure S10B). Box plot analysis of the nascent RNA transcript levels demonstrated that the INTS6-dependent increase in DARTs levels was the most significant at DSBs (Figure 6B). Additionally, we generated a merged metagene plot showing nascent RNA levels, SETX-ChIP-seq and DRIP-seq for cut *Asi*SI sites (Figure 6C), as well as for uncut sites (Figure 6D), using a 2.5 kb frame size on both sides of DSBs. Interestingly, the metagene profile of SETX occupancy correlated with DARTs that were increased in an INTS6-dependent manner. Furthermore, the metagene profile of increased DARTs and SETX negatively correlated with the DRIP profile in the control sample, suggesting that INTS6 depletion might lead to the accumulation DNA:RNA hybrids around DSBs (Figure 6C–F). Similar overlapping metagene patterns were also observed at DSBs near sites with high or low transcriptional activity and HR or NHEJ-prone sites (Supplementary Figure S11A–D).

These findings support the notion that INTS6-dependent DARTs could contribute to DNA:RNA hybrids located at DSBs. INTS6, by binding to DNA:RNA hybrids, subsequently recruits SETX, which, in turn, resolves them. These results underscore the pivotal role of INTS6-mediated DNA:RNA hybrid autoregulation.

### INTS6 is required for efficient DNA damage repair

**Figure 7.**
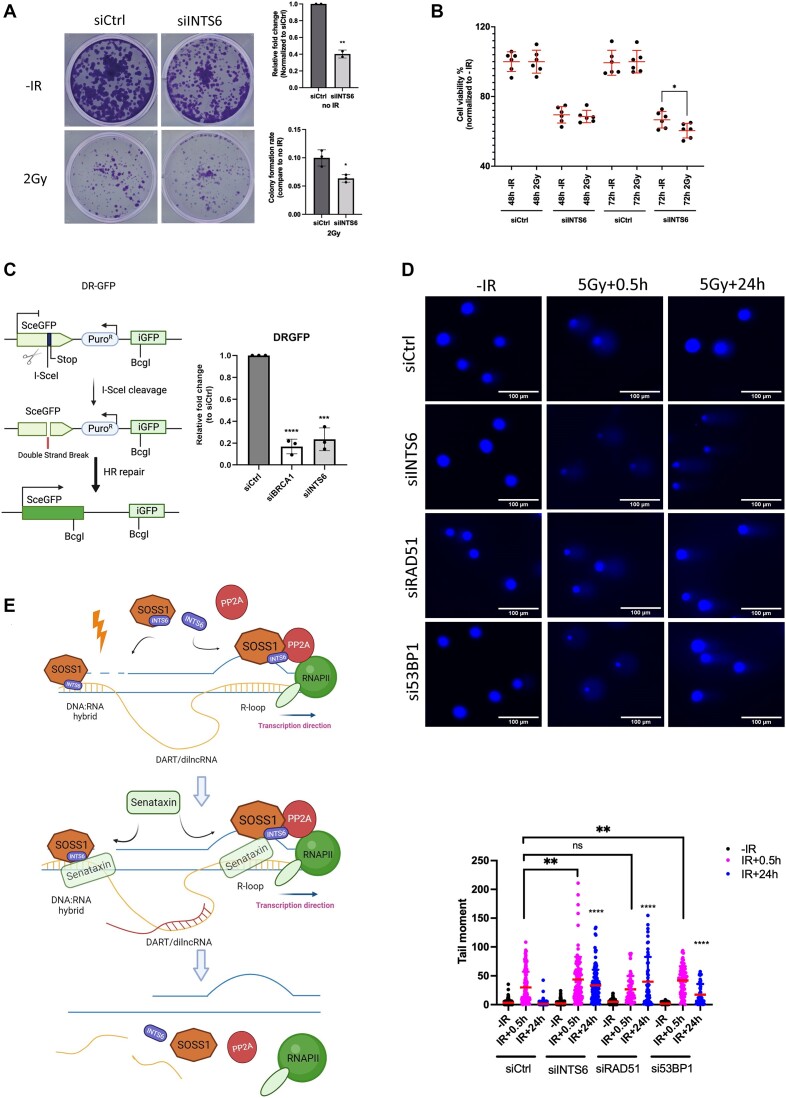
INTS6 is required for efficient DNA damage repair. (**A**) Left: Representative images of the clonogenic assay in control and INTS6 knockdown cells. The cells were stained and counted after 10 days of growing. Right: Quantification of left. * *P* ≤ 0.05, **P ≤ 0.01. (**B**) MTT assay to show cell viability (%) at indicated time points in siCtrl and siINTS6 cells with or without 2 Gy IR treatment. Error bar = mean ± SD, significance was determined using unpaired Student’s *t*-test, *P ≤ 0.05. (**C**) Left: Drawing of DR-GFP HR reporter strategy. Right: Bar chart shows the efficiency of HR repair in DR-GFP HeLa reporter cells, as measured by FACS. BRCA1 knockdown was used as the positive control. ****P ≤ 0.0001, ***P ≤ 0.001. (**D**) Top: Representative images of comet assay in siCtrl, siINTS6, siRAD51 and si53BP1 cells. siRAD51 and si53BP1 cells were used as positive controls. IR = 5 Gy. Error bar = 100 μm. Bottom: Quantification of top. ****P ≤ 0.0001, **P ≤ 0.01. (**E**) Model: INTS6 as part of tetrameric SOSS1 complex binds to DNA:RNA hybrids at DSBs and recruits PP2A to dephosphorylate RNAPII. Depletion of INTS6 results in increased levels of DARTs and DNA:RNA hybrids. INTS6 interacts with SETX and is required for its recruitment to damaged sites. SETX, in turn, resolves DNA:RNA hybrids at DSBs facilitating their INTS6-dependent autoregulation. Image was created with Biorender.

Intrigued by these findings, we sought to validate the significance of INTS6 in DDR. Initially, we conducted a clonogenic assay in control and INTS6 knockdown cells exposed to IR. The results revealed a growth defect attributed to INTS6 depletion, which was further exacerbated by IR treatment, although the additive effect was only mild (Figure 7A). To support these data, we also performed an MTT assay to measure cell viability in control and INTS6 depleted cells after exposure to IR. Indeed, we detected a mild but significant growth defect at 72 h post-IR in cells lacking INTS6 (Figure 7B). To investigate the specific DSB repair pathway in which INTS6 might be involved, we utilized reporter cell lines. The DR-GFP HR HeLa reporter cell line contains a specially designed *Sce*GFP sequence, with an I-*Sce*I cutting site, a stop codon and an in-frame GFP template. Transient expression of the pCBA*Sce*I plasmid allows measurement of GFP-expressing cells by FACS to assess HR repair efficiency. Depletion of INTS6 in this system resulted in robust HR inhibition, nearly reaching the level observed upon BRCA1 depletion, which served as a positive control (Figure 7C). Additionally, we used the EJ5 NHEJ HeLa reporter system, in which the disrupted GFP is reactivated by the NHEJ process. Again, a significant reduction in NHEJ efficiency was observed after INTS6 depletion (BAY-8400, a DNA-PK inhibitor, was used as the positive control) (Supplementary Figure S12A). Furthermore, a comet assay, which visualizes broken DNA ends, revealed a significant delay in DNA repair in INTS6-depleted cells compared to control cells. The extent of delayed repair in INTS6 knockdown cells was comparable to the level of damage in RAD51- or 53BP1-deficient cells, which were used as controls (Figure 7D and Supplementary Figure S12B). To exclude the possibility that a reduced level of repair proteins in reporter cell lines upon INTS6 depletion was the cause of the observed phenotype, we tested the protein levels of selected DDR factors by western blot. Neither RPA32 nor RAD51 was affected by INTS6 depletion suggesting, that the repair defects were directly caused by INTS6 depletion (Supplementary Figure S12C). Collectively, these data indicate that INTS6 is crucial for efficient DNA repair.

Based on our findings, we propose (Figure 7E) that INTS6, as part of the tetrameric SOSS1 complex, binds to DNA:RNA hybrids at DSBs and recruits PP2A to dephosphorylate RNAPII. Depletion of INTS6 results in increased levels of DARTs and DNA:RNA hybrids. INTS6 interacts with SETX and is required for its recruitment to damaged sites. SETX, in turn, resolves DNA:RNA hybrids at DSBs, facilitating their INTS6-dependent autoregulation.

## Discussion

Transcription by RNAPII is a highly regulated process that is essential for cellular function. The Integrator complex, conserved across metazoans (31), exerts control over the fate of many nascent RNAs transcribed by RNAPII. With inherent RNA endonuclease activity, Integrator contributes to the biogenesis of small nuclear RNAs and enhancer RNAs. Notably, the Integrator complex initiates premature transcription termination of various protein-coding genes, leading to the attenuation of gene expression. Consequently, the Integrator complex plays a crucial role in shaping the transcriptome, ensuring robust gene inducibility when needed (31,50,62). When coupled with PP2A phosphatase, the Integrator complex forms the Integrator–PP2A complex, regulating the dephosphorylation of active RNAPII (36), which enables the conformational shift to an active/open state, causing the collapse of the transcription bubble and the release of DNA from RNAPII (37).

Although the molecular structure of the Integrator has been solved by Cryo-EM (36,63), showing the exact interaction architecture of its subunits, evidence suggests different interaction patterns exist between various subunits. This indicates they might co-exist and form subcomplexes that function outside of the Integrator complex. A recent Cryo-EM study reveals the structures of two sub-complexes: INTS10/13/14/15 and INTS5/8/10/15 (64). Additionally, INTS3 and INTS6 can interact as part of the SOSS1 complex (40). The SOSS1 complex, a multiprotein entity, is vital in the DDR, functioning downstream of the MRN complex to promote DNA repair and the G2/M checkpoint. Essential for efficient HR-dependent repair of DSBs and ataxia telangiectasia mutated (ATM)-dependent signalling pathways, the SOSS1 complex acts as a sensor of single-stranded DNA, particularly binding to polypyrimidines (65). Moreover, the trimeric SOSS1 complex promotes phase separation at DSBs (38).

This study reveals (Figure 7E) that in response to DNA damage, an increased amount of INTS6 binds to trimeric SOSS1 to form the tetrameric SOSS1 complex. This tetrameric SOSS1 then recruits PP2A to DSBs, facilitating the dephosphorylation of RNAPII. Transcription at DSBs is crucial for efficient DNA repair, but it eventually needs to be terminated. The termination process, associated with the interaction of RNAPII and the cleavage/polyadenylation complex, relies on the S2P CTD of RNAPII. The release of mRNA and the initiation of a new cycle for subsequent rounds of transcription require dephosphorylation of the CTD of RNAPII. Thus, the INTS6-dependent recruitment of PP2A can be seen as a prerequisite for efficient transcription termination at DSBs. Interestingly, a recent structural study (37) found that, in the absence of damage, the Integrator–PP2A complex exists with INTS6 blocking the PP2A phosphatase active site in an inactive closed conformation. When the Integrator–PP2A complex encounters paused RNAPII, it rearranges its structure and incorporates with the pre-elongation complex (PEC) to form a PEC-Integrator–PP2A complex, which dephosphorylates RNAPII CTD, then terminates transcription.

Furthermore, our study finds that INTS6 interacts with and recruits SETX to DSBs. SETX, recently identified as a transcription termination factor in mammalian cells, suggests a dual role for INTS6 in the regulating transcription termination at DSBs (45).

DNA:RNA hybrids and R-loops are recognized as potential threats to genome stability (22,29,30). However, they are closely associated with transcription at DSBs and can serve as binding platforms for numerous DDR factors (12,24–28). Our study unveils the ability of INTS6 to bind to DNA:RNA hybrids, a crucial step for its localization to DSBs. Depletion of INTS6 results in a significant increase in levels of nascent transcripts at DSBs, known as DARTs. As DARTs are produced as single-stranded RNA molecules, they can hybridize with the DNA overhang post-resection, forming DNA:RNA hybrid and to the exposed ssDNA behind paused RNAPII, to create R-loops. Notably, within 1 kb on each side of the DSB, the enriched INTS6-mediated transcripts overlap with DNA:RNA DRIP-seq peaks and SETX ChIP-seq peaks. SETX, a well-known DNA:RNA helicase, resolves DNA:RNA hybrids at DSBs (22). This study uncovers a previously unknown interaction between INTS6 and SETX, shedding light on the SOSS1-dependent resolution of DNA:RNA hybrids and their autoregulation.

Overall, we demonstrate (Figure 7E) the increased formation of a tetrameric SOSS1 complex, comprising INTS6 and the trimeric SOSS1, in response to DNA damage. INTS6’s specific binding to DNA:RNA hybrids plays a pivotal role in recruiting PP2A to DSBs and facilitating the dephosphorylation of RNAPII. Depletion of INTS6 leads to the accumulation of DARTs and the stabilization of DNA:RNA hybrids at DSB sites. Additionally, INTS6 interacts with and mediates the recruitment of SETX to DSBs, facilitating the resolution of DNA:RNA hybrids. These findings underscore the critical role of the SOSS1 complex in autoregulating DNA:RNA hybrid dynamics and promoting effective DNA repair.

## Supplementary Material

gkae937_Supplemental_File

## Data Availability

Data reported in this paper can be shared by the lead contact upon request. Mass spectrometry proteomics data have been deposited to the ProteomeXchange Consortium via PRIDE (https://www.ebi.ac.uk/pride/) under project accession number PXD048694. chrRNA-seq data have been deposited to GEO (https://www.ncbi.nlm.nih.gov/geo/) and can be accessed under GSE246729. Any additional information required to re-analyze the data reported in this work paper is available from the lead contact upon request.
