# Supplementary material for: Tetrameric INTS6-SOSS1 complex facilitates DNA:RNA hybrid autoregulation at double-strand breaks
Source: Nucleic Acids Res. 2024 Oct 24;52(21):13036–56. doi: 10.1093/nar/gkae937 (PMC11602137; doi:10.1093/nar/gkae937)
Supplement: gkae937_Supplemental_File [file gkae937_supplemental_file.pdf]

Supplementary Figure 1

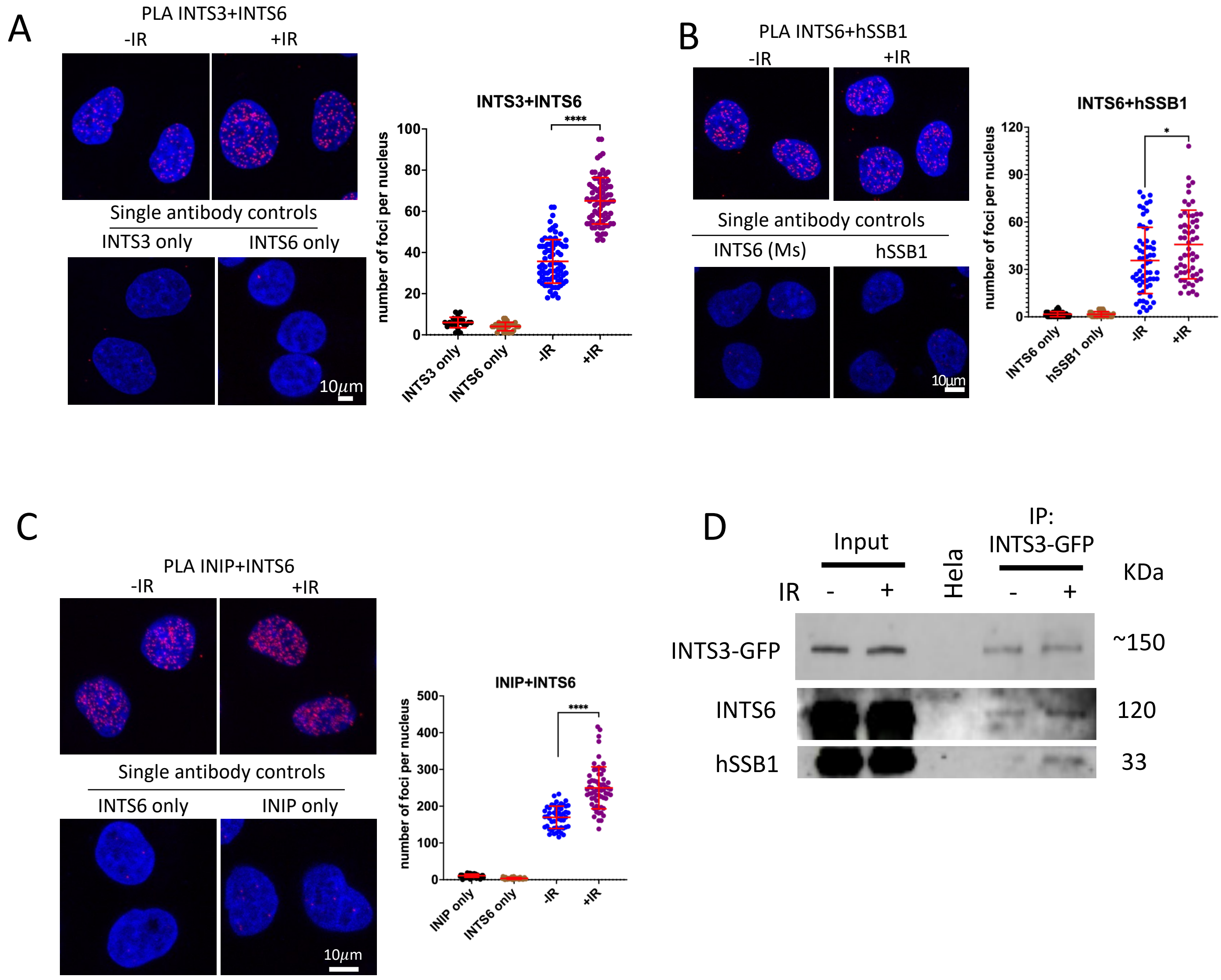

Supplementary Figure 2

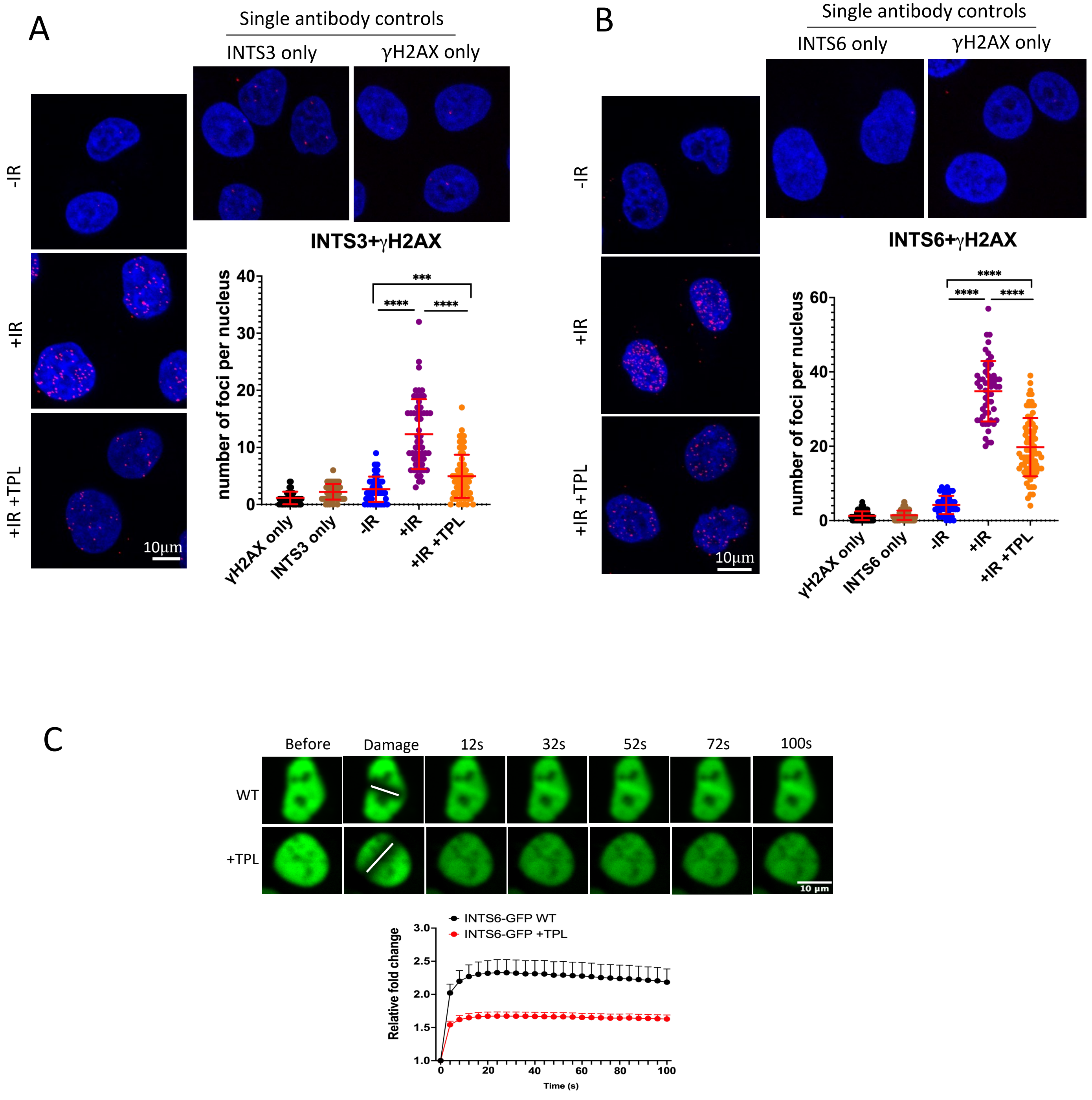

Supplementary Figure 3

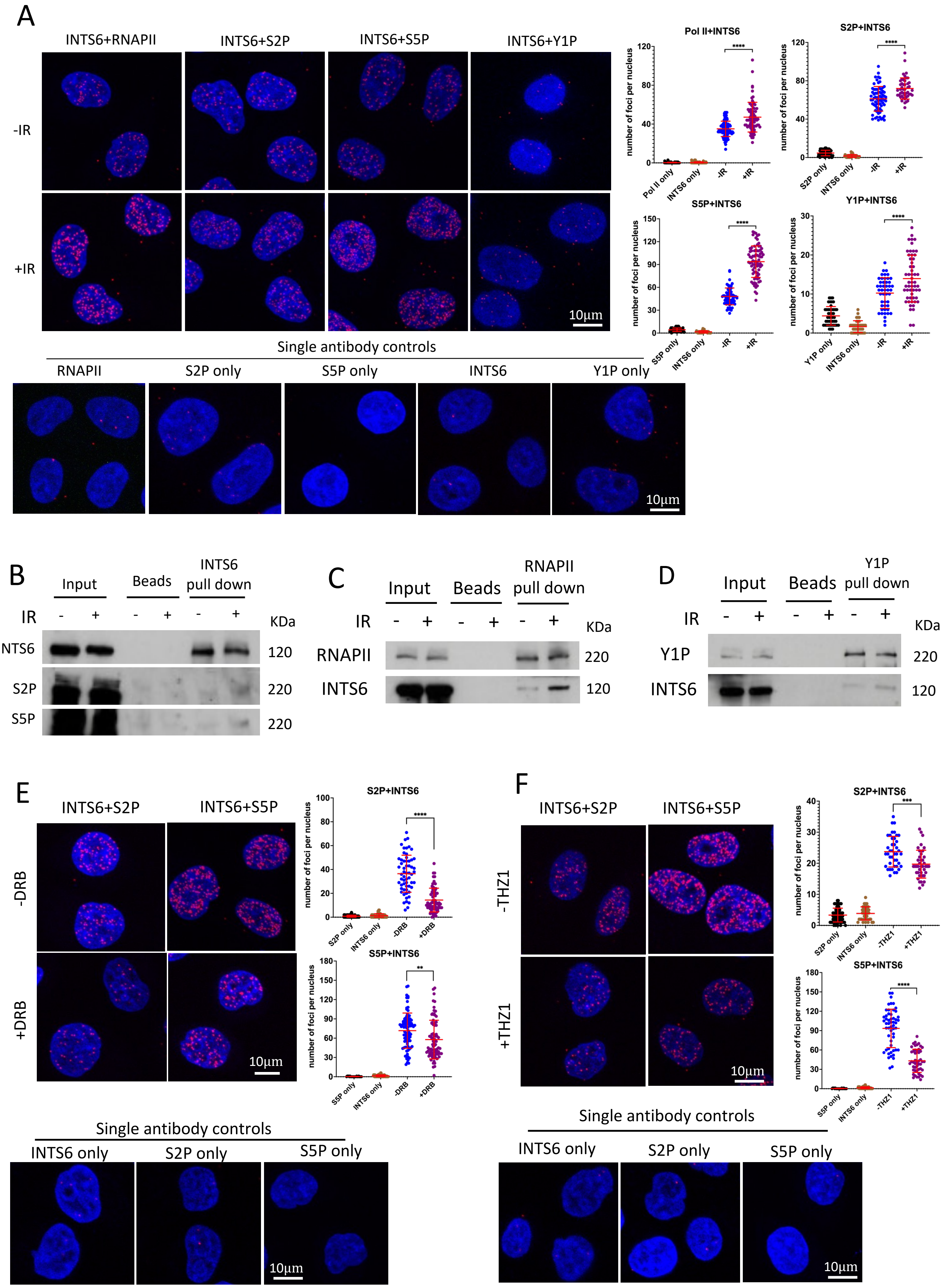

Supplementary Figure 4

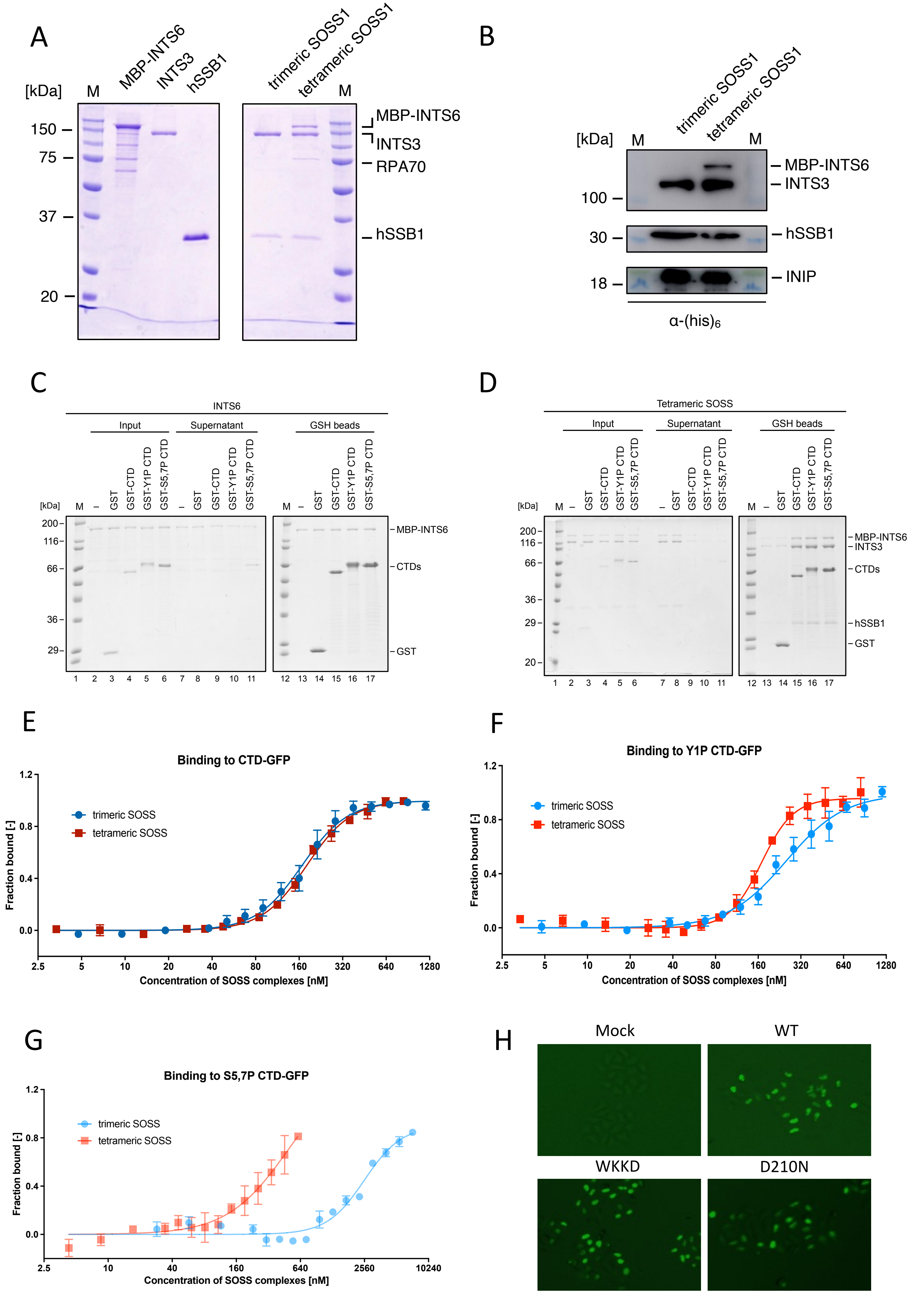

Supplementary Figure 5

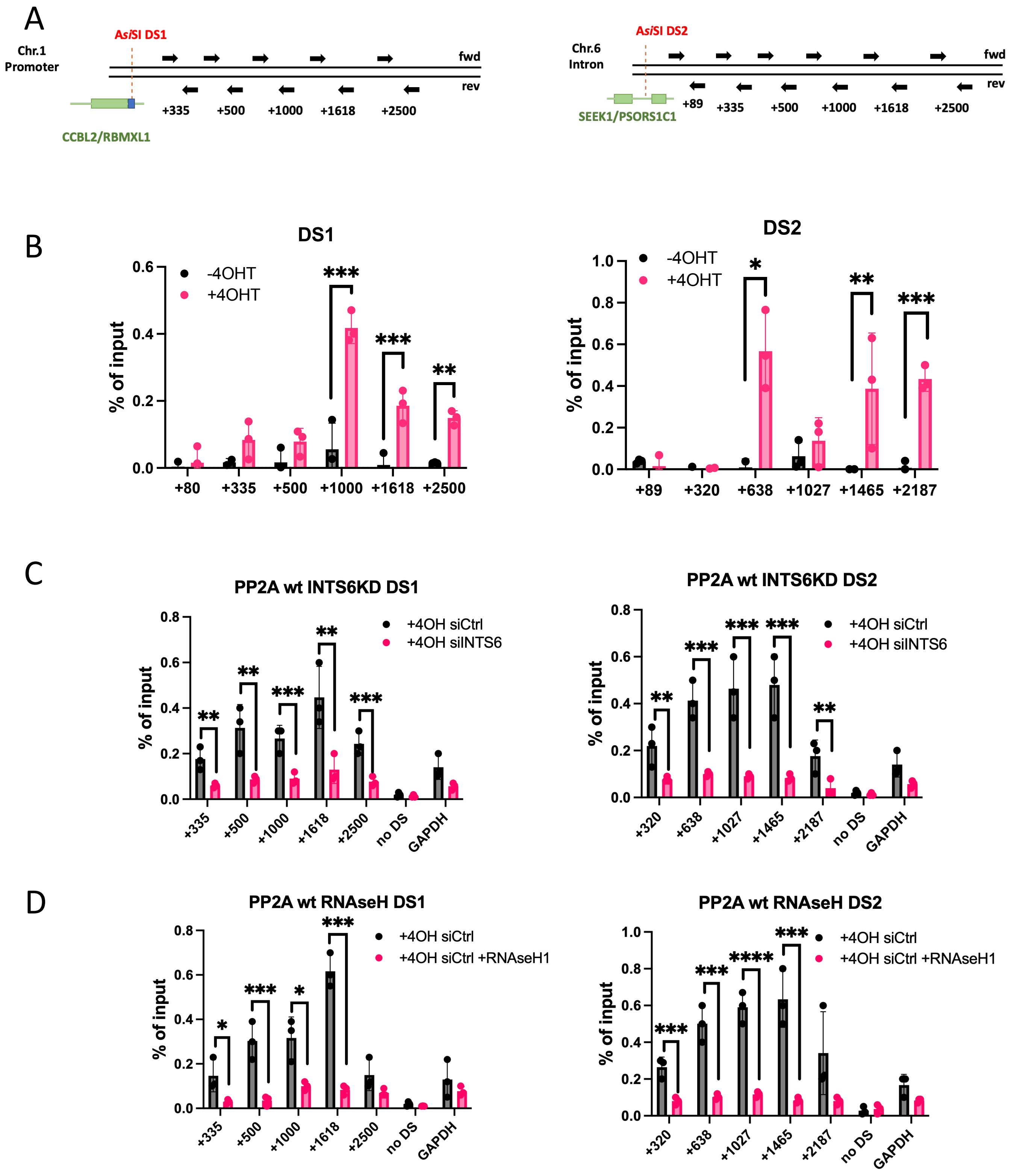

Supplementary Figure 6

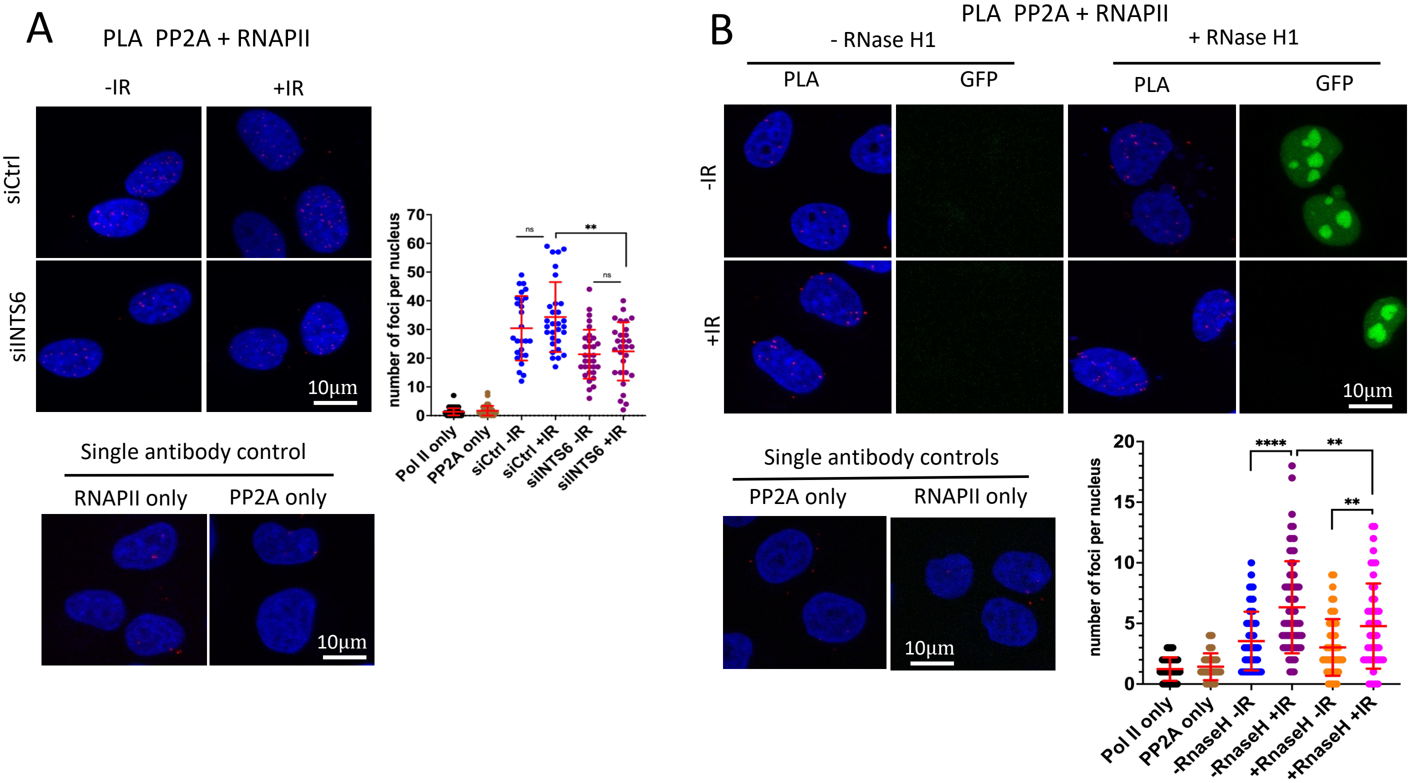

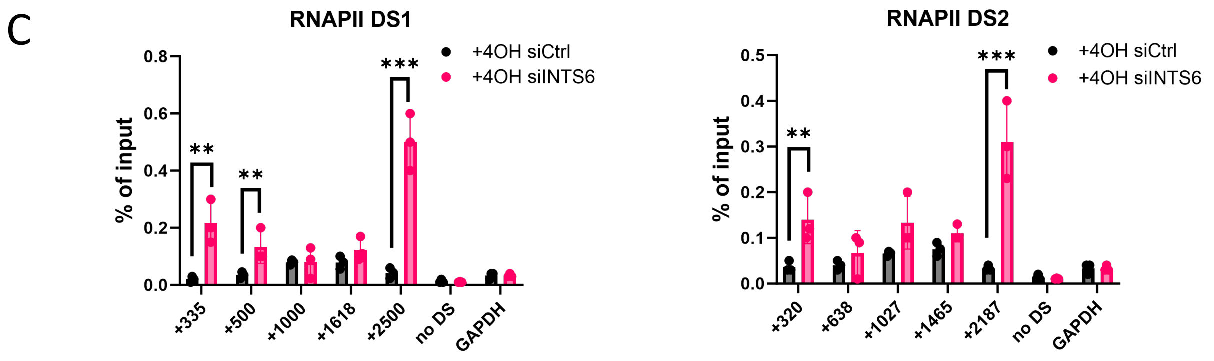

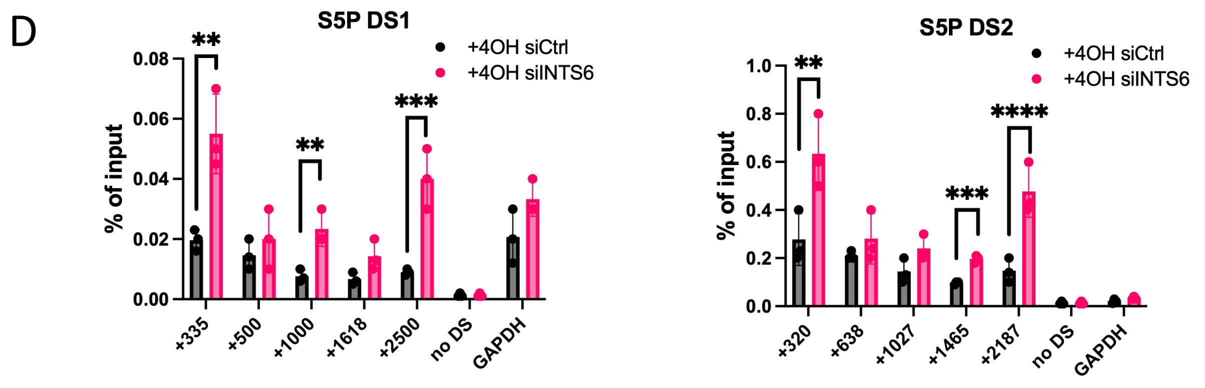

Supplementary Figure 7

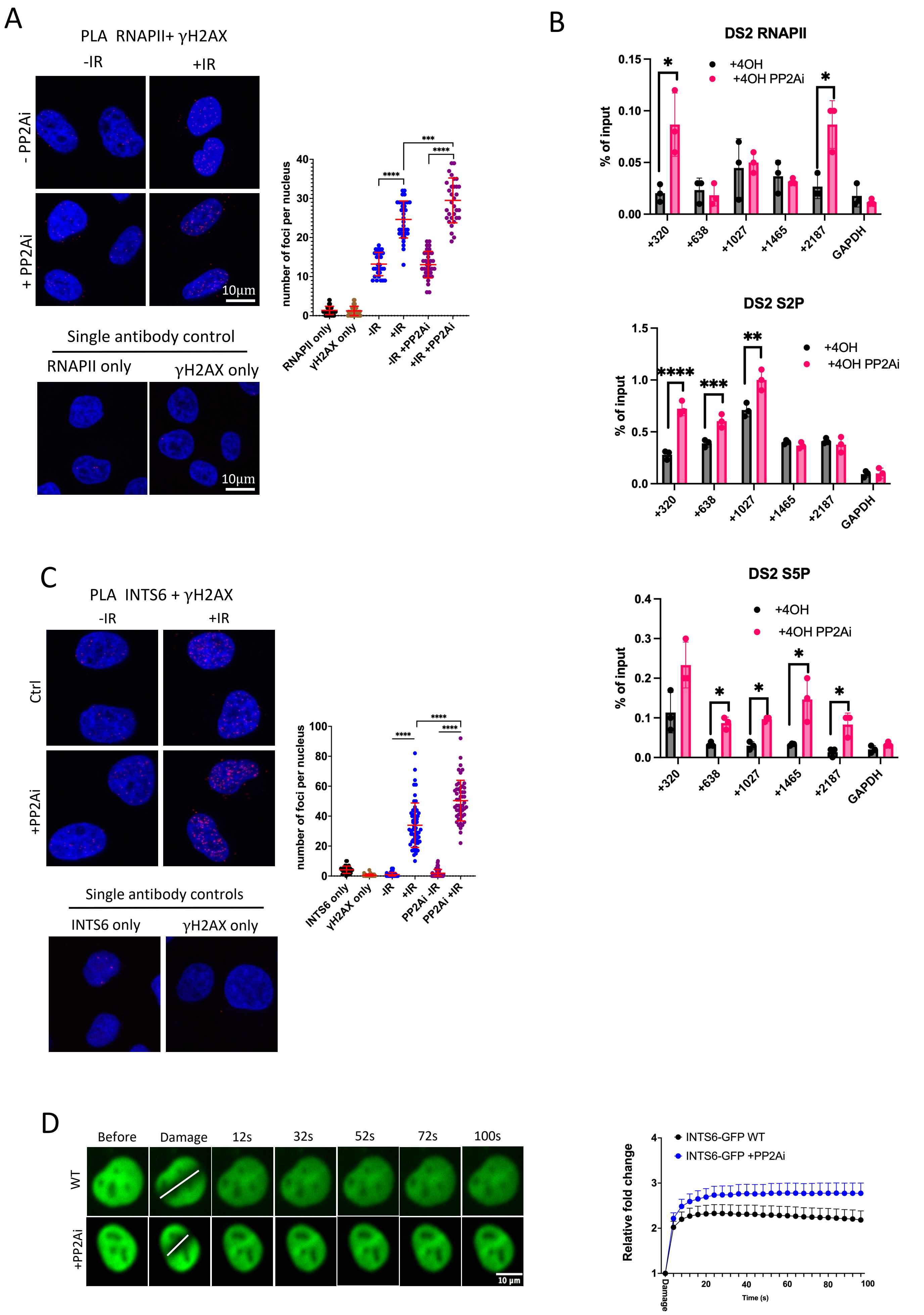

Supplementary Figure 8

A

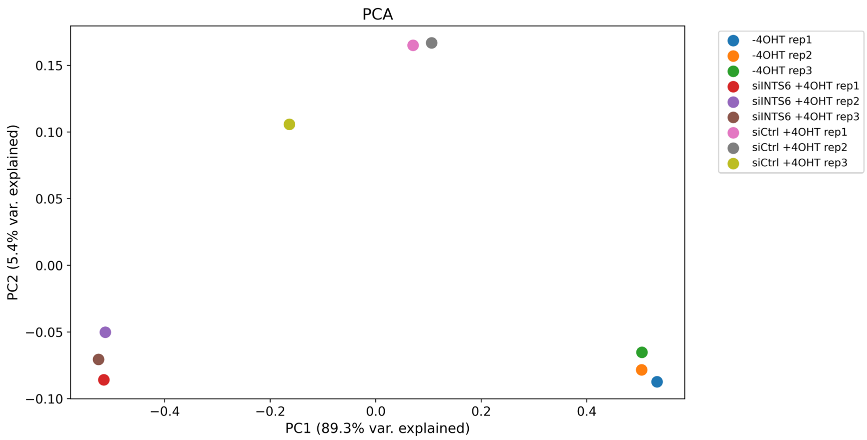

B

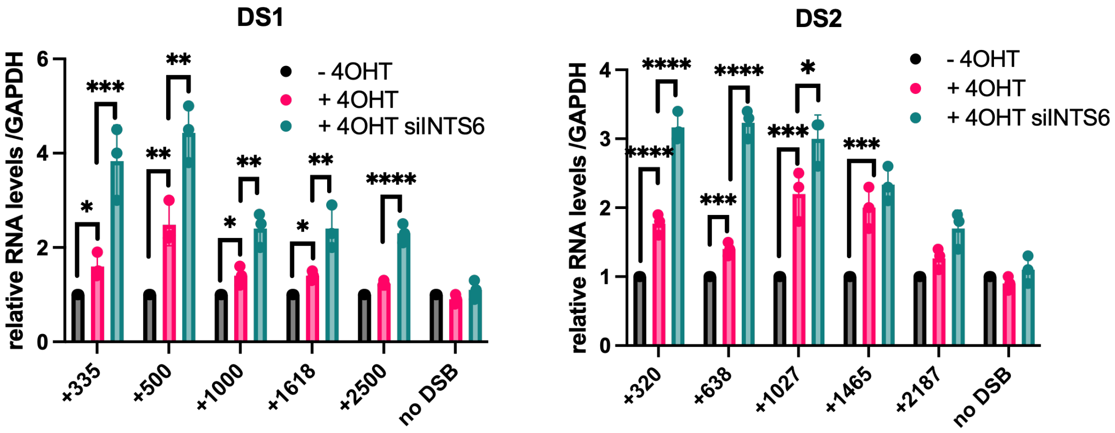

C

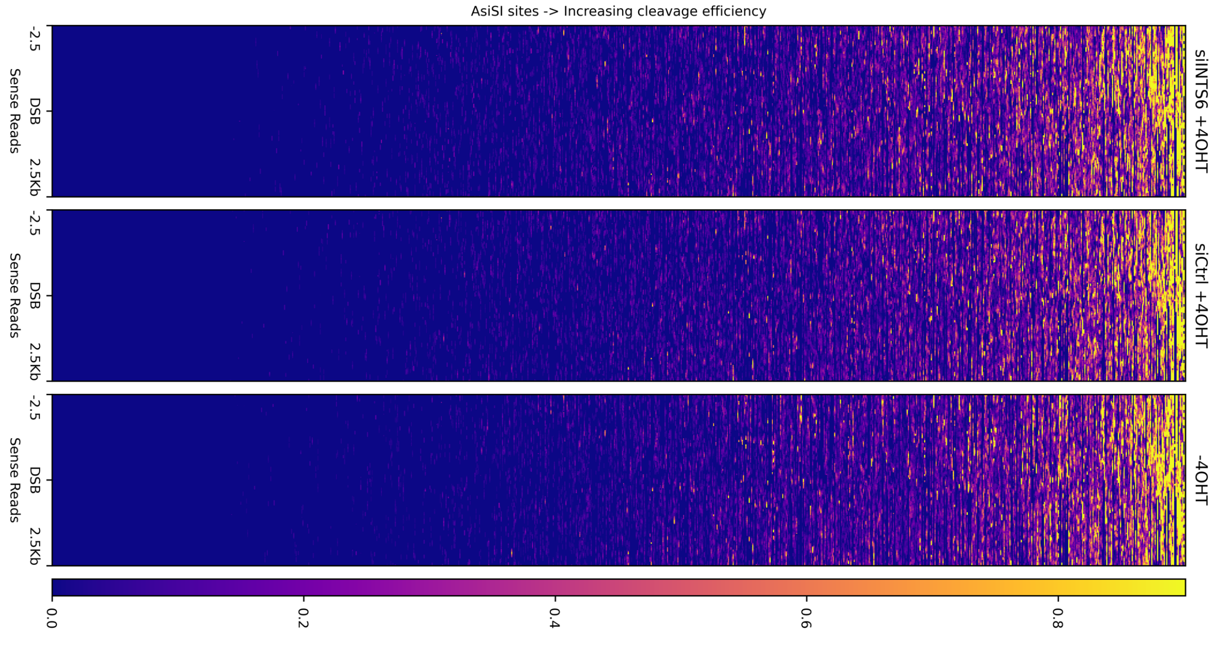

Supplementary Figure 9

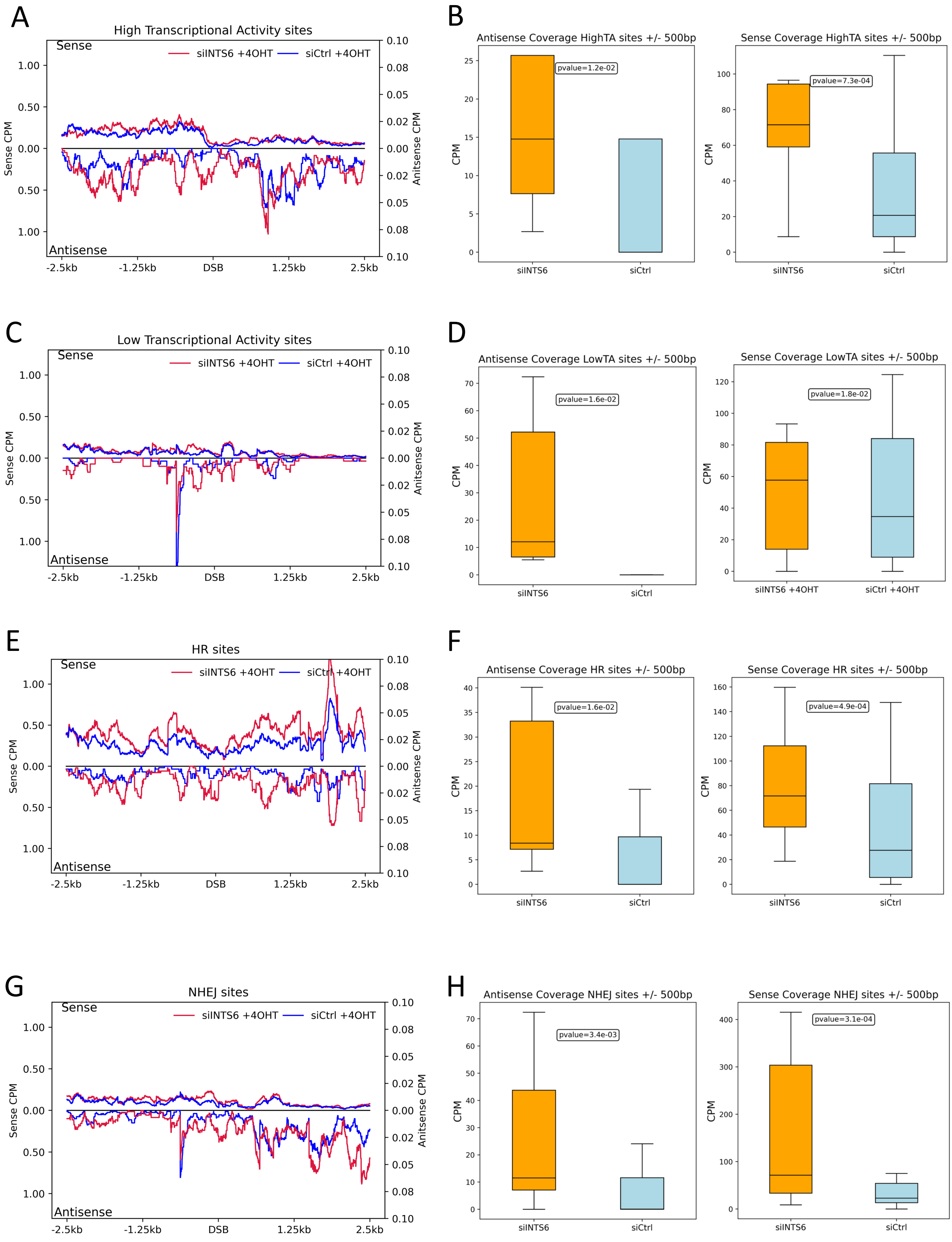

Supplementary Figure 10

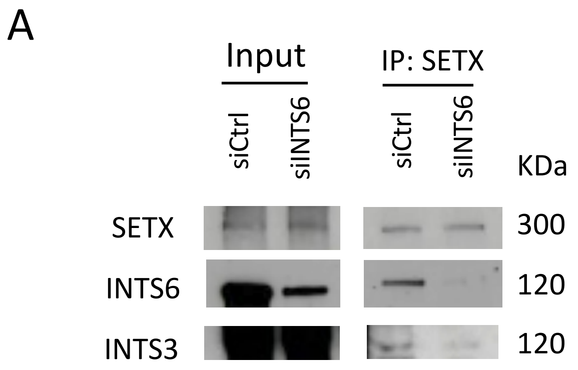

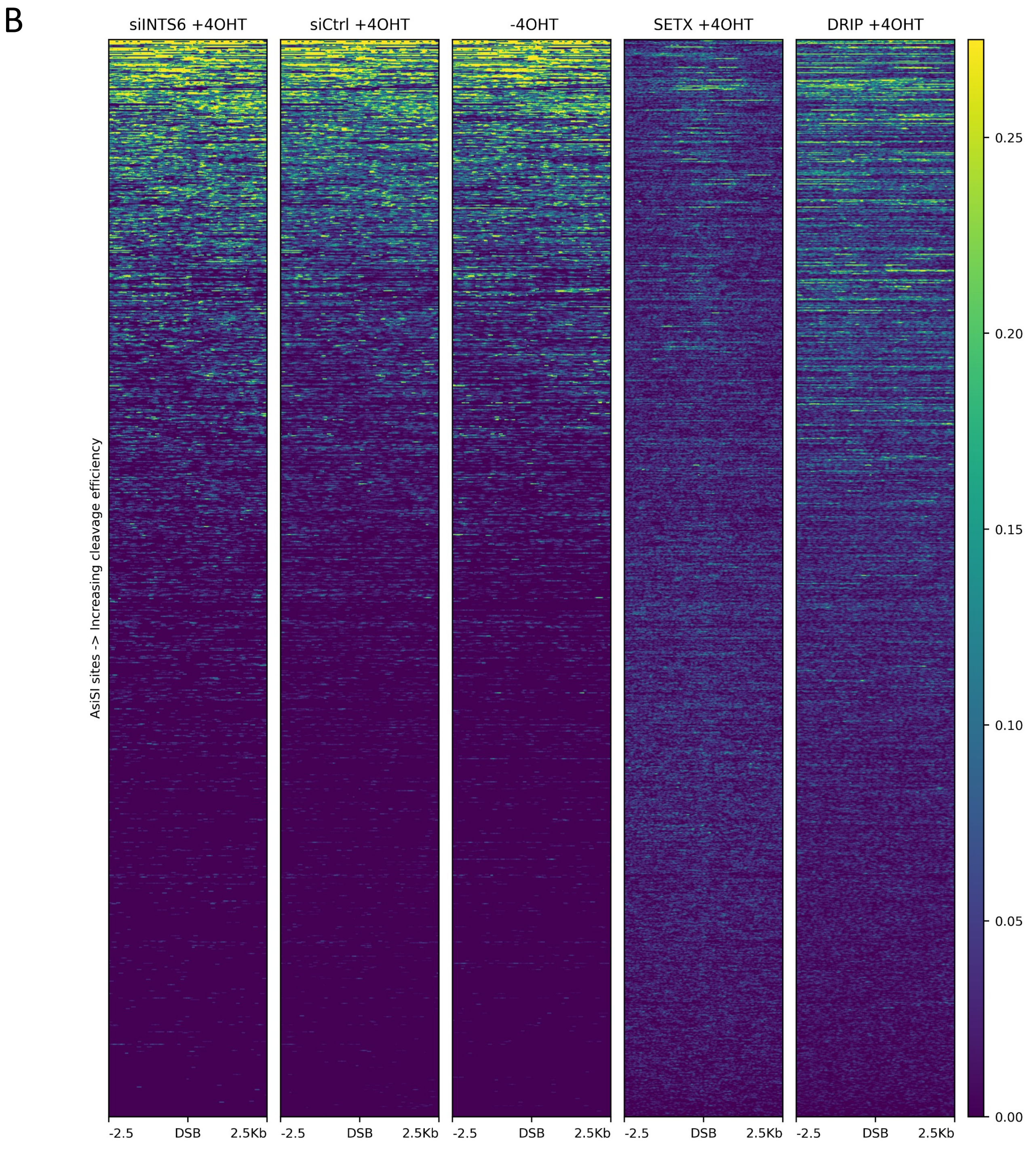

Supplementary Figure 11

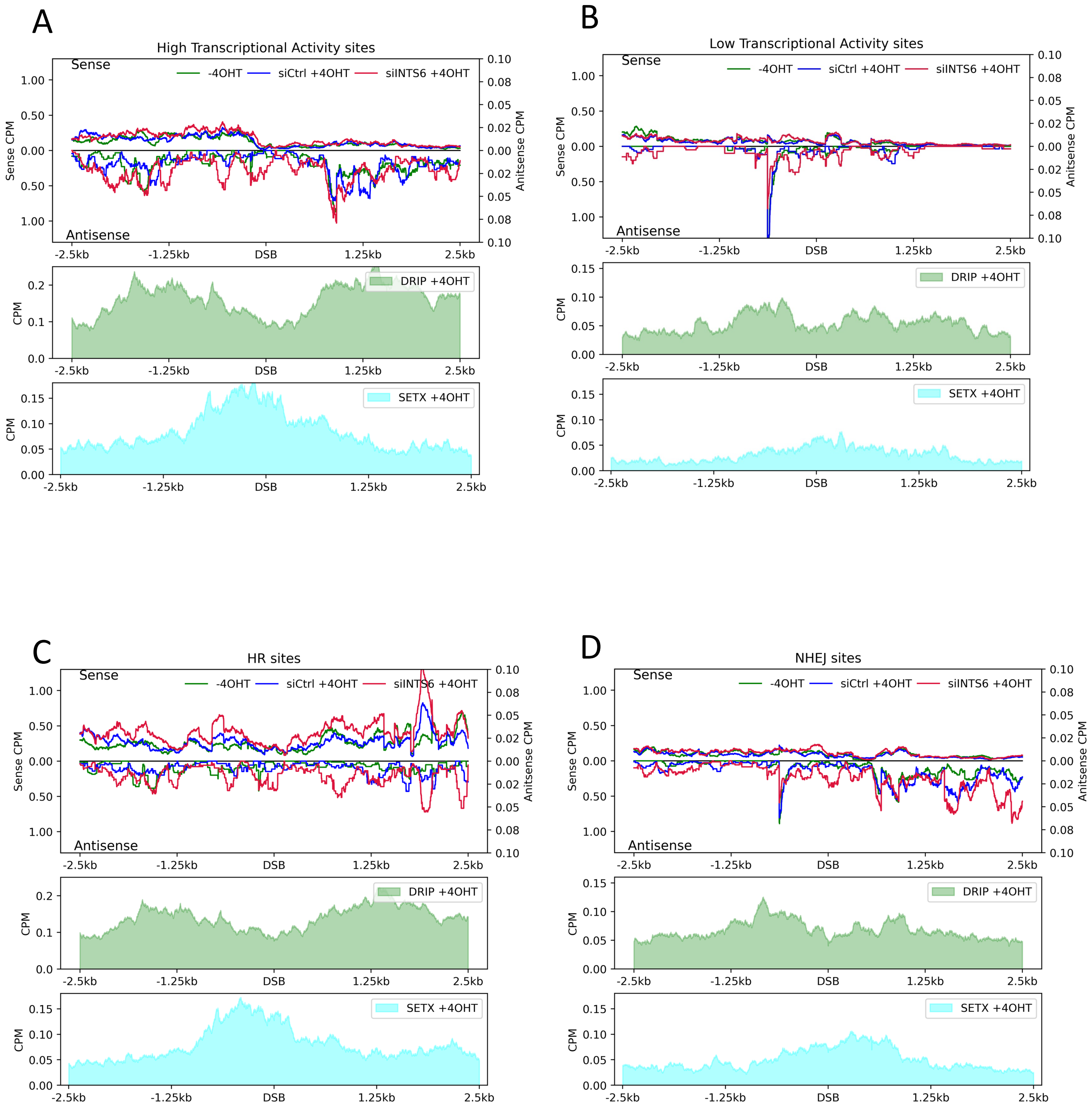

Supplementary Figure 12

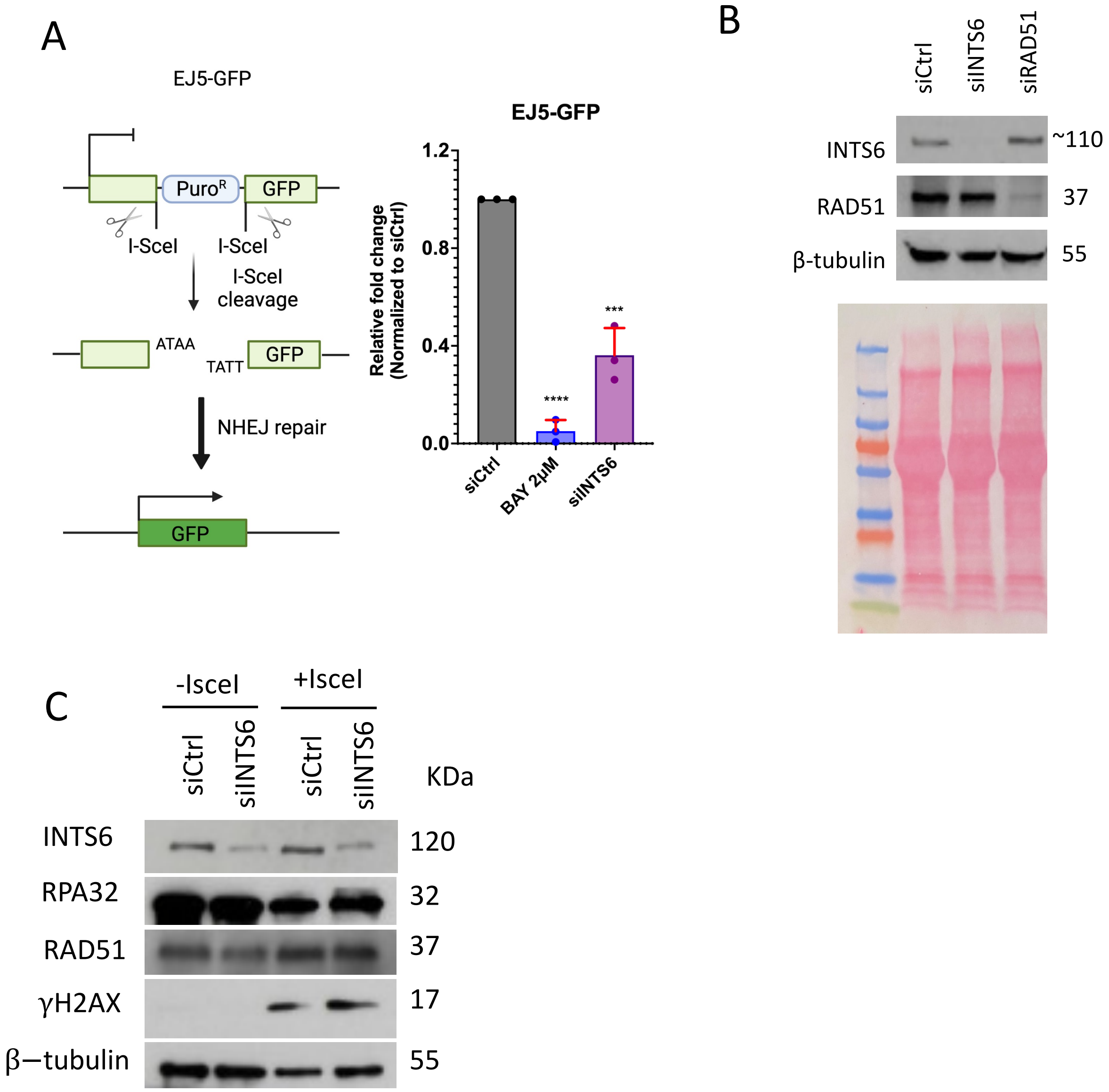

Supplementary Figure legend

Supplementary Figure S1

A-C) PLA of INTS3 and INTS6 (A), INTS6 and hSSB1(B), INTS6 and INIP (C) in cells with or without IR. IR=10Gy. Samples are collected 10 min post IR. Left: representative confocal microscopy images; Right: quantification of top, error bar = mean ± SD, significance was determined using non-parametric Mann-Whitney test. *p ≤ 0.05, ****p ≤ 0.0001. Scale bar =10μm. Single antibodies were used as a negative control.

D) Immunoprecipitation of GFP from cells transiently express INTS3-GFP with or without IR treatment (IR=10Gy, samples are collected 10 min post IR), followed by western blot showing signals for INTS3-GFP, INTS6 and hSSB1. KDa: size of the proteins.

Supplementary Figure S2

A-B) PLA of INTS3 (A) or INTS6 (B) and γH2AX in cells in the presence or absence of Triptolide (20 μM, 1h) with or without IR treatment. IR=10Gy. Samples are collected 10 min post IR. Left: representative confocal microscopy images; right: quantification of left, error bar = mean ± SD, significance was determined using non-parametric Mann-Whitney test. ***p ≤ 0.001, ****p ≤ 0.0001. Scale bar =10μm. Single antibodies were used as a negative control.

C) Laser striping of cells transiently expressing INTS6-GFP plasmid in the presence or absence of Triptolide (20 μM, 1h). Representative confocal microscopy images and quantification (n ≥ 20) show GFP signal at the indicated time points. Error bars, mean ± SEM.

Supplementary Figure S3

A) PLA of INTS6 and RNAPII or S2P or S5P or Y1P with or without IR. IR=10Gy. Samples are collected 10 min post IR. Left: representative confocal microscopy images; right: quantification of top, error bar = mean ±SD, significance was determined using non-parametric Mann-Whitney test. ****p ≤ 0.0001. Scale bar =10μm. Single antibodies were used as a negative control.

B-D) Immunoprecipitation of endogenous INTS6 (B), RNAPII (C) and Y1P (D) from cells with or without IR treatment (IR=10Gy, samples are collected 10 min post IR), followed by western blots. KDa: size of the proteins.

E) PLA of S2P or S5P and INTS6 with or without 100μM DRB treatment for 2h. IR=10Gy. Samples are collected 10 min post IR. Left: representative confocal microscopy images; right: quantification of left, error bar = mean ± SD, significance was determined using non-parametric Mann-Whitney test. **p ≤ 0.01, ****p ≤ 0.0001. Scale bar =10μm. Single antibodies were used as a negative control.

F) PLA of S2P or S5P and INTS6 in cells with or without 1μM THZ1 treatment for 2h. IR=10Gy. Samples are collected 10 min post IR. Left: representative confocal microscopy images; right: quantification of left, error bar = mean ± SD, significance was determined using non-parametric Mann-Whitney test. ***p ≤ 0.001, ****p ≤ 0.0001. Scale bar =10μm. Single antibodies were used as a negative control.

Supplementary Figure S4

A) A representative 15% SDS-PAGE gel depicting purified components of SOSS1 complexes (INIP is not visible due to its size).

B) 18% SDS-PAGE followed by western blot probing for components of trimeric or tetrameric SOSS1 complexes.

C) A representative SDS-PAGE gel depicting *in vitro* pull-down assay of INTS6 with GST-tagged CTD, GST-tagged CTD modified on tyrosine 1 (Y1P), or GST-tagged CTD modified on serine 5 and 7 (S5,7P).

D) A representative SDS-PAGE gel depicting *in vitro* pull-down assay of tetrameric SOSS1 complex with GST-CTD, GST-Y1P-CTD or GST-S5,7P-CTD.

E-G) Microscale-thermophoresis (MST) binding curves of trimeric and tetrameric SOSS1 complexes with unmodified CTD-GFP (E), Y1P-CTD-GFP (F) or S5,7P-CTD-GFP (G). Measured in triplicates and the lines represent the Hill fit.

H) Confocal microscopy images showing GFP expression of transiently transfected RNAseH1 $^{wt\text{-}GFP}$ or RNAseH1 $^{WKKD\text{-}GFP}$ (binding and catalytic) or RNAseH1 $^{D210N\text{-}GFP}$ (catalytic) mutants. Mock is used as a negative control.

Supplementary Figure S5

A) Drawing of ChIP probes positions around DS1 and DS2.

B) Bar chart showing γH2AX ChIP signals around DS1 and DS2 in the absence or presence of 4-OHT. n=3, significance was determined by student t-test, ***p ≤ 0.001, **p ≤ 0.01, *p ≤ 0.05.

C) Bar charts showing PP2A ChIP signals at DS1 and DS2 in siCtrl and siINTS6 cells. $n \geq 3$. Error bar = mean ± SD, significance was determined using unpaired Students t-test, $**p \leq 0.01$, $***p \leq 0.001$.

D) Bar charts showing PP2A ChIP signals at DS1 and DS2 in cells with RNaseH1 overexpression. $n \geq 3$. Error bar = mean ± SD, significance was determined using unpaired Students t-test, $*p \leq 0.05$, $***p \leq 0.001$.

Supplementary Figure S6

A) PLA of PP2A and RNAPII with or without IR in the control or INTS6 knockdown cells. IR=10Gy. Samples are collected 10 min post IR. Left: representative confocal microscopy images; right: quantification of left, error bar = mean ± SD, significance was determined using non-parametric Mann-Whitney test. $**p \leq 0.01$. Scale bar =10μm. Single antibodies were used as a negative control.

B) PLA of PP2A and RNAPII with or without IR in cells in the presence or absence of RNaseH1 treatment. IR=10Gy. Samples are collected 10 min post IR. Left: representative confocal microscopy images; right: quantification of left, error bar = mean ± SD, significance was determined using non-parametric Mann-Whitney test. $**p \leq 0.01$, $****p \leq 0.0001$. Scale bar =10μm. Single antibodies were used as a negative control.

C-D) Bar charts showing RNAPII (C) and S5P (D) ChIP signals at DS1 and DS2 in siCtrl and siINTS6 cells. $n \geq 3$. Error bar = mean ± SD, significance was determined using unpaired Students t-test, $**p \leq 0.01$, $***p \leq 0.001$.

Supplementary Figure S7

A) PLA of RNAPII and γH2AX with or without IR in the presence or absence of PP2A inhibitor (LB-100, 2.5μM, 2h). IR=10Gy. Samples are collected 10 min post IR. Left: representative confocal microscopy images; right: quantification of left, error bar = mean ± SD, significance was determined using non-parametric Mann-Whitney test. $****p \leq 0.0001$. Scale bar =10μm. Single antibodies were used as a negative control.

B) Bar charts showing RNAPII, S2P and S5P ChIP signals at DS2 in the absence or presence of PP2A inhibitor (LB-100, 2.5μM, 4h). $n \geq 3$. Error bar = mean ± SD, significance was determined using unpaired Students t-test, $**p \leq 0.01$, $*p \leq 0.05$.

C) PLA of INTS6 and γH2AX with or without IR in the presence or absence of PP2A inhibitor (LB-100, 2.5μM, 2h). IR=10Gy. Samples are collected 10 min post IR. Left: representative confocal microscopy images; right: quantification of left, error bar = mean ± SD, significance

was determined using non-parametric Mann-Whitney test. ****$p \leq 0.0001$. Scale bar =10µm. Single antibodies were used as a negative control.

D) Laser striping of cells transiently expressing INTS6-GFP plasmid in the presence or absence of PP2A inhibitor (LB-100, 2.5µM, 2h). Representative confocal microscopy images and quantification ($n \geq 20$) show GFP signal at the indicated time points. Error bars, mean ± SEM.

Supplementary Figure S8

A) PCA plots show a comparison of chrRNA-Seq coverage in the 5kb flanking region of cut *Asi*SI sites (details in supplementary Table S4) between sample replicates in INTS6 knockdown and control conditions.

B) Bar chart showing the manual ChromeRNA-qPCR signals around DS1 and DS2 in cells absence or presence of 4-OHT/INTS6. n=3, significance was determined by student t-test, *$p \leq 0.05$, **$p \leq 0.01$, ***$p \leq 0.001$.

C) Heatmaps show sense nascent RNA (chrRNA-seq) read coverage across annotated AsiSI sites (details in supplementary Table S4) sorted based on cleavage efficiency.

Supplementary Figure S9

A) Metagene plots showing chrRNA-Seq sense and antisense coverage in INTS6 knockdown and control cells with damage induction (4-OHT) around 2.5kb flank region of highly transcriptionally active *Asi*SI sites (n=20).

B) Box plot shows chrRNA Seq antisense (Left) and sense (Right) coverage in +/- 500 bp flank of highly transcriptionally active *Asi*SI sites (n=20).

C), As in A) for transcriptionally inactive *Asi*SI sites (n=20)

D) As in B) for transcriptionally inactive *Asi*SI sites (n=20). In box plots, the bar represents the median, while the top and bottom edges correspond to the 75th and 25th percentiles. In this case, all three values are zero, indicating no antisense reads in the Ctrl for LowTA sites.

E) As in A) for HR prone *Asi*SI sites (n=30)

F) As in B) for HR prone *Asi*SI sites (n=30)

G) As in A) for NHEJ prone *Asi*SI sites (n=30)

H) As in B) for NHEJ prone *Asi*SI sites (n=30)

Supplementary Figure S10

A) Immunoprecipitation of endogenous SETX from siCtrl and siINTS6 cells with IR treatment (IR=10Gy, samples are collected 10 min post IR), followed by western blots. KDa: size of the proteins.

B) Heatmaps show the siINTS6+4OHT chrRNA-Seq coverage, siCtrl+4OHT chrRNA-Seq coverage, -4OHT chrRNA-Seq coverage, SETX+4OHT ChIP-Seq coverage and S9.6+4OHT DRIP-Seq coverage across all *Asi*SI cut sites sorted by cleavage efficiency. The reference genome is human hg19.

Supplementary Figure S11

A) Metagene plots showing DRIP and SETX ChIP coverage upon damage induction along with sense and antisense coverage of chrRNA-Seq with damage induction around 2.5kb flank region of highly transcriptionally active *Asi*SI sites (n=20). The reference genome is human hg19.

B) Metagene plots showing DRIP and SETX ChIP coverage upon damage induction along with sense and antisense coverage of chrRNA-Seq with damage induction around 2.5kb flank region of transcriptionally inactive *Asi*SI sites (n=20). The reference genome is human hg19.

C) Metagene plots showing DRIP and SETX ChIP coverage upon damage induction along with sense and antisense coverage of chrRNA-Seq with damage induction around 2.5kb flank region of HR-prone *Asi*SI sites (n=30). The reference genome is human hg19.

D) Metagene plots showing DRIP and SETX ChIP coverage upon damage induction along with sense and antisense coverage of chrRNA-Seq with damage induction around 2.5kb flank region of NHEJ-prone *Asi*SI sites (n=30). The reference genome is human hg19.

Supplementary Figure S12

A) Left: Drawing of EJ5-GFP NHEJ reporter strategy. Right: Bar chart showing the efficiency of NHEJ repair in EJ5 HeLa reporter cells, as measured by FACS. BAY (selective DNA-PK inhibitor) was used as the positive control.

B) Western blot shows the knockdown efficiency of INTS6 and RAD51 in HeLa cells.

C) Western blot shows the protein expression in siCtrl and siINTS6 DRGFP cells with or without IsceI plasmid. KDa: size of proteins.

Table S1. List of oligonucleotides used in this study for *in vitro* work.

Name	Sequence 5'-3' Supplementary table	Use
pR209	TACTTCCAATCCAATCGATGACGACGGAGACCTTTG	Amplification of INTS6
pR210	TTATCCACTTCCAATGTTATTACTAATTGCTATTAATATGGTTGATC	Amplification of INTS6
pR211	GAACAGTTGACAGGTGTGCC	Sequencing of INTS6
pR212	TCCAGTCCTTCTTCCCCTCT	Sequencing of INTS6
pR213	CAAATGGGGAACTACCAGGA	Sequencing of INTS6
Part of the following substrates: RNA:DNA-hybrid, R-loop		
pR219	GGGTGAACCTGCAGGTGGGCGGCTGCTCATCGTAGGTTAGTTGGTAGAATTCGGCAGCGTC	EMSA experiments
Part of the following substrate: R-loop substrate for EMSA experiments		
pR223	AAAGAUGUCCUAGCAAGGCAC	EMSA experiments
Part of the following substrates: RNA:DNA-hybrid, R-loop		
pR630	[Cy3]-GTGCCTTGCTAGGACATCTTT	EMSA experiments

Table S2. List of oligonucleotides used in this study for CHIP and DRIP experiments.

Primer Name	Primer Sequence (5'-3')
DS1-335 fwd	GAATCGGATGTATGCGACTGATC
DS1-335 rev	TTCCAAAGTTATTCCAACCCGAT
DS1-500 fwd	CCTGGATATGAGTTTGATCAGC
DS1-500 rev	CTCTCCTTTCGCTGACACTG
DS1-1000 fwd	AGGAATTGACTGCGGTGTTC
DS1-1000 rev	GGGGAGGAGGAAAGGTGTAG
DS1-1618 fwd	TGAGGAGGTGACATTAGAACTCAGA
DS1-1618 rev	AGGACTCACTTACACGGCCTTT
DS1-2500 fwd	GCCATAACAGAGGGTGGAAA
DS1-2500 rev	AACTTTAGGATGGGGCTGCT
DS2-320 fwd	CTAGGTCTGGCTCCTCCTGA
DS2-320 rev	CTCCCTGAACCGCCTAGAAC
DS2-638 fwd	GCTGCCTGAGATGCCTGTAA

DS2-638 rev	ATCCAGACAGGCTCCTCCTC
DS2-1027 fwd	CCCCAGCTCCTTAACACAGA
DS2-1027 rev	CATGGTGCAGAAGGTGCATT
DS2-1465 fwd	AGCCCAGTGGCACAGGAATA
DS2-1465 rev	CCTTCAGGGGTGACACATCAG
DS2-2187 fwd	AGGAATCCACCTATCCGCCT
DS2-2187 rev	GGCTAACAGACTTCCAGGCA
GAPDH fwd	AACCTGCCAAATATGATGAC
GAPDH rev	AGGAAATGAGCTTGACAAAG
no DSB-fwd	ATTGGGTATCTGCGTCTAGTGAGG
no DSB-rev	GACTCAATTACATCCCTGCAGCT

Table S3. List of oligonucleotides used in this study for Gibson cloning.

Primer Name	Primer Sequence (5'-3')
INTS6_EGFP_fwd	Cagctgttgggctcgcggttatgcccatcttactgttcctg
INTS6_EGFP_rev	Ctcacagagcctccaccccccattgctattaatatggttgatctgattg
BACKBONE_EGFP_fwd	Tcaaccatattaatagcaatggggggtggaggctctgtgag
BACKBONE_EGFP_rev	Aggaacagtaagatgggcataaccgcgagcccaacagctg

Table S4. List of AsiSI cut sites used for metagene plots and downstream analysis.

	Chr	Start	End	SITE
1	chr1	9649445	9649452	SITE495
2	chr1	40974643	40974650	SITE516
3	chr1	89458596	89458603	SITE526
4	chr1	224032647	224032654	SITE566
5	chr2	43358338	43358345	SITE702
6	chr2	68384748	68384755	SITE709
7	chr2	74734761	74734768	SITE713
8	chr2	85822593	85822600	SITE716
9	chr2	120124565	120124572	SITE722
10	chr2	208030727	208030734	SITE742
11	chr3	52232162	52232169	SITE765
12	chr3	98618164	98618171	SITE778
13	chr4	83934286	83934293	SITE840
14	chr4	178363575	178363582	SITE871
15	chr5	68462850	68462857	SITE899
16	chr5	79784139	79784146	SITE903
17	chr5	142785049	142785056	SITE919
18	chr6	27145366	27145373	SITE949
19	chr6	31105427	31105434	SITE953
20	chr6	37321811	37321818	SITE956

21	chr6	49917582	49917589	SITE961
22	chr6	90348186	90348193	SITE970
23	chr6	135819347	135819354	SITE974
24	chr6	144607568	144607575	SITE975
25	chr6	149888105	149888112	SITE976
26	chr7	75807506	75807513	SITE1025
27	chr7	92861490	92861497	SITE1031
28	chr7	99679507	99679514	SITE1034
29	chr8	66546347	66546354	SITE1093
30	chr8	116680631	116680638	SITE1107
31	chr8	124781209	124781216	SITE1110
32	chr9	29212799	29212806	SITE1123
33	chr9	36258513	36258520	SITE1129
34	chr9	130693170	130693177	SITE1159
35	chr9	130889407	130889414	SITE1160
36	chr10	3110977	3110984	SITE4
37	chr10	94051014	94051021	SITE40
38	chr11	24518475	24518482	SITE72
39	chr11	75525760	75525767	SITE101
40	chr12	13154717	13154724	SITE133
41	chr12	22093988	22093995	SITE136
42	chr12	130091880	130091887	SITE173
43	chr13	114894658	114894665	SITE204
44	chr14	54955825	54955832	SITE219
45	chr17	61850855	61850862	SITE380
46	chr17	80250840	80250847	SITE392
47	chr18	7566712	7566719	SITE396
48	chr18	19320804	19320811	SITE404
49	chr19	2456093	2456100	SITE432
50	chr19	42497855	42497862	SITE474
51	chr20	1207615	1207622	SITE582
52	chr20	20032924	20032931	SITE594
53	chr20	32032086	32032093	SITE601
54	chr20	37360268	37360275	SITE605
55	chr20	42087117	42087124	SITE608
56	chr21	33245518	33245525	SITE622
57	chr21	46221789	46221796	SITE628
58	chr22	20850307	20850314	SITE649
59	chr22	38864101	38864108	SITE657
60	chrX	1510671	1510678	SITE1176
61	chrX	53111426	53111433	SITE1191

Table S5. List of reagents used in this study.

REAGENT or RESOURCE	SOURCE	IDENTIFIER
Antibodies		
INTS6	Abcam	ab86369
DICE1 (H-6)	Santa Cruz	sc-376524
ANTI-DNA-RNA HYBRID, CLONE S9.6	Sigma	MABE1095
phospho-Histone H2A.X (Ser139)	Sigma	05-636
Phospho-gamma-H2AX (Ser139)	Invitrogen	MA533062
PPP2R1A	Bethyl	A300-962A
RNA Polymerase II RPB1-8WG16	Biolegend	664912
RNA polymerase II CTD repeat YSPTSPS (phospho S5)	Abcam	ab5131
RNA polymerase II CTD repeat YSPTSPS (phospho S2)	Abcam	ab5095
RNA polymerase II CTD repeat YSPTSPS	Abcam	ab26721
Senataxin	Nouvs Bio	NBP1-94712
INTS3	bethyl	A302-051A
INTS3	Proteintech	16620-1-AP
hSSB1	Bethyl	A301-938A
hSSB1	Lifespan BioSciences	LS-C173584
INIP	Novus Biologicals	NBP1-81682
GFP	Proteintech	3h9-100
AbFlex® RNA Pol II CTD phospho Tyr1	Active Motif	92129
RAD51	N/A	FE lab homemade
beta-tubulin	Abcam	ab6046
RPA32/RPA2 antibody [9H8]	Abcam	ab2175
Bacterial and virus strains		
NEB® 5-alpha Competent E. coli (High Efficiency)	New England Biology	C2987H
MAX Efficiency™ DH10bac Competent Cells	ThermoFisher	10361012
Chemicals, peptides, and recombinant proteins		
Triptolide	Enzo life science	BV-1761-1
5,6-Dichloro-1-beta-D-ribofuranosylbenzimidazole (DRB)	Cayman Chemical	10010302
THZ1	Stratech Scientific	A8882-APE-10mM
LB-100	Stratech Scientific	B4846-APE-5mg
(Z)-4-hydroxy Tamoxifen (4-OHT)	Cayman Chemical	14854-1mg-CAY
BAY-8400	Cambridge Bioscience	HY-132293-1mg
Critical commercial assays		
Duolink® In Situ Red Starter Kit Mouse/Rabbit	Sigma	DUO92101-1KT
Experimental models: Cell lines		
HeLa	ATCC	N/A
DRGFP HeLa	MG lab	N/A

EJ5 HeLa	MG lab	N/A
U2OS	GL lab	N/A
U2OS AsiSI-ER	GL lab	N/A
Oligonucleotides		
siControl (ON-TARGETplus, Dharmacon SMARTpool)	Dharmacon	D-001810-03-05
siBRCA1(ON-TARGETplus, Dharmacon SMARTpool)	Dharmacon	J-003461-09-0005
siINTS6(ON-TARGETplus, Dharmacon SMARTpool)	Dharmacon	L-012417-00-0005
si53BP1(ON-TARGETplus, Dharmacon SMARTpool)	Dharmacon	M-003548-01-0005
siRAD51 #1*	IDT	5' GACUGCCAGGAUAAAGCUU 3'
siRAD51 #2*	IDT	5' GUGCUGCAGCCUAAUGAGA 3'
*Use both siRAD51 #1 and siRAD51 #2 together to do transient knock down.		
Recombinant DNA: Plasmids		
pCBASceI	Addgene	Plasmid #26477
INTS6-GFP	this study	N/A
pRNH1^{WT}-GFP	NJP Lab	N/A
pRNH1^{D210N}-GFP	NJP Lab	N/A
pRNH1^{WKKD}-GFP	NJP Lab	N/A
pFRT-TODestRFP_RNAseH1	Addgene	Plasmid #65785
Software and algorithms		
GraphPad Prism 9	GraphPad Software, San Diego, California USA, www.graphpad.com	N/A
Fiji	(Schindelinet et al)	N/A
CellProfiler	(Carpenter et al)	N/A
BioRender	https://www.biorender.com/	N/A
